# Synthesis of novel ligands targeting phenazine biosynthesis proteins as a strategy for antibiotic intervention

**DOI:** 10.1007/s00706-017-2100-z

**Published:** 2017-11-30

**Authors:** Nikolaus Guttenberger, Thomas Schlatzer, Mario Leypold, Sebastian Tassoti, Rolf Breinbauer

**Affiliations:** Institute of Organic Chemistry, Graz University of Technology, Graz, Austria

**Keywords:** Antibiotics, Enzymes, Etherification, Inhibitor, Phenazines

## Abstract

**Abstract:**

In this contribution, we report synthetic strategies towards potential ligands for the study of binding differences between PhzE, the first enzyme in the biosynthesis of phenazines, and the related enzyme anthranilate synthase. The ligands were designed with the overriding goal to develop new antibiotics via downregulation of phenazine biosynthesis.

**Graphical abstract:**

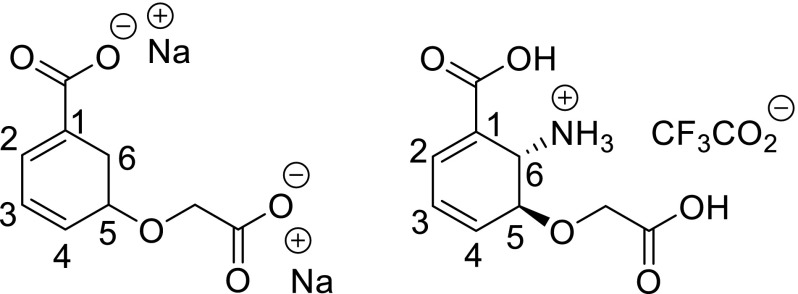

**Electronic supplementary material:**

The online version of this article (10.1007/s00706-017-2100-z) contains supplementary material, which is available to authorized users.

## Introduction

Phenazines are redox-active secondary metabolites mainly produced by bacteria such as *Streptomyces* and *Pseudomonas* conferring the producer a competitive advantage over other microorganisms [1, 2]. Amongst other modes of action, phenazines are able to reduce molecular oxygen for the generation of reactive oxygen species (ROS) [3], and facilitate energy generation [4–6], e.g., in biofilms [1]. The production of these secondary metabolites has been shown to extend the lifespan of the producing organism markedly, making the phenazine biosynthesis an attractive strategy for the development of new antibiotics [1, 2].

Over the last decade, the biosynthesis of phenazines has been elucidated in considerable detail [1, 7, 8], yet there are still gaps in understanding. Chorismic acid, an important intermediate of the shikimate pathway, is the starting material of the core phenazine biosynthesis. Five enzymes, namely PhzA/B, PhzD, PhzE, PhzF, and PhzG encoded in the *phz* operon, catalyze the transformation of chorismic acid into 5,10-dihydrophenazine-1,6-dicarboxylic acid (DHPDC) and 5,10-dihydrophenazine-1-carboxylic acid (DHPCA). Both compounds are central intermediates in the biosynthesis of strain-specific phenazines (Scheme 1) [1, 7].

**Figure:**
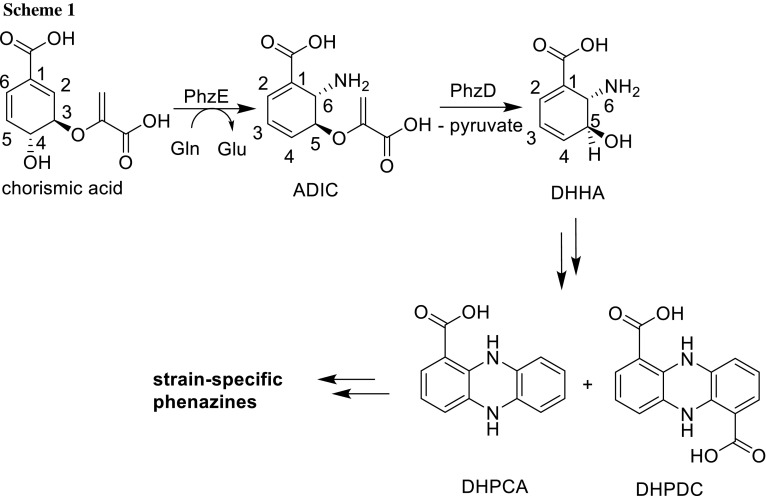

So far, only ligands for PhzA/B have been developed, which interfere with the later stage of phenazine biosynthesis [9–11]. However, strategically it would be more attractive to inhibit the phenazine biosynthesis at the earliest possible stage for effective antibiotic intervention. Upon inhibition of PhzE, phenazine production would be disabled leading to a loss of competitive advantage of phenazine-producing bacteria. Therefore, the synthesis of potent ligands interfering with PhzE would possess attractive potential for the development of new antibiotics.

In the first step of the phenazine biosynthesis, PhzE transforms chorismic acid into 2-amino-2-deoxyisochorismic acid (ADIC) [12]. The same step is catalyzed by AdsX in the biosynthesis of tilimycin as well as tilivalline [13], and putatively by TomD in tomaymycin biosynthesis [14]. Homodimeric PhzE consists of two domains, namely the GATase domain, where NH_3_ is produced, and a “menaquinone, siderophore, tryptophan (MST)” domain where the reaction of chorismic acid to ADIC takes place [12]. NH_3_ is channeled into the MST domain via a tunnel with a length of 25 Å. Within the MST domain, the ammonia reacts at the *Si*-face of the C-6 of chorismic acid to yield ADIC. [12]. An enzyme related to PhzE is anthranilate synthase (AS) [15–17]. In AS, ADIC is converted to anthranilic acid, whereas in PhzE ADIC is handed over to PhzD for the synthesis of strain-specific phenazines (Scheme 2).

**Figure:**
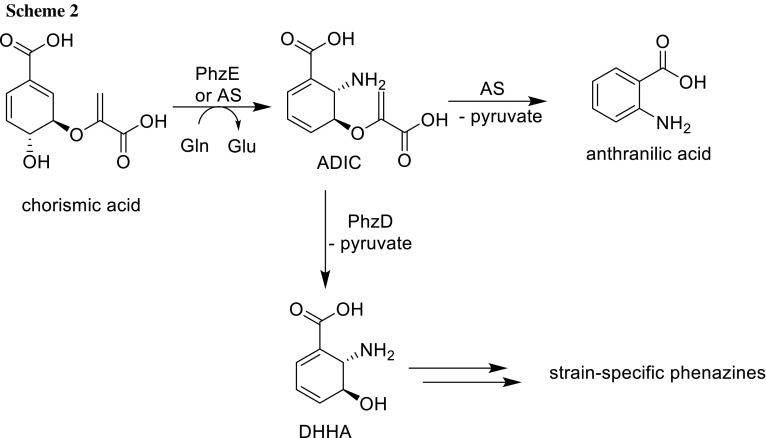

This discrepancy is somewhat puzzling, as only three amino acid residues, namely Ser^217^, Ser^368^, and Thr^369^ that are present in the first coordination sphere of chorismic acid in PhzE, differ in AS [12]. It is assumed that the different mode of action originate either from the time ADIC resides in the active site or from an altered release path [12]. In addition, it remains unclear why in crystallization experiments of PhzE benzoate and pyruvate instead of chorismate or ADIC were found in the active site of the MST domain [12]. It was hypothesized that the instability [18] of ADIC could account for this observation [12]. Our goal is to study binding differences between PhzE and AS to develop specific PhzE inhibitors as antibiotics. In this paper, we describe our synthetic efforts leading to the preparation of two potential ligands of PhzE via two independent routes, which will help to pursue this goal. Importantly, we could demonstrate that late-stage structural modifications are feasible in one of the two ligand syntheses that will give access to additional ligands in the future.

## Results and discussion

**Fig. 1:**
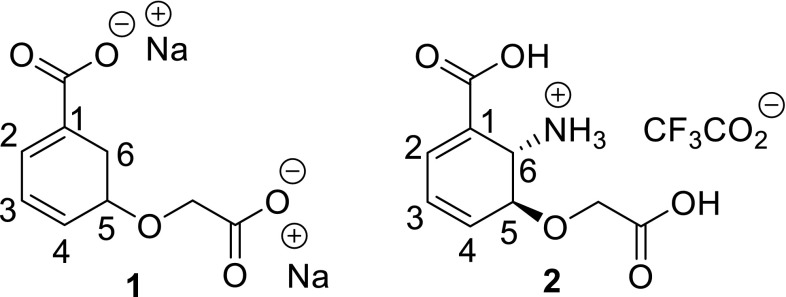
Ligands for the study of binding differences between PhzE and AS

We designed ligands *rac*-5-(carboxylatomethoxy)cyclohexa-1,3-dienecarboxylate disodium salt (**1**) and *rac*-*trans*-6-amino-5-(carboxymethoxy)cyclohexa-1,3-dienecarboxylic acid TFA salt (**2**) which are structurally similar to the products of PhzE and AS enzymes, but should not be turned over by these enzymes as the labile enol ether moiety is replaced by a stable alkyl ether group (Fig. 1).

Ligand **1** is based on chorismate as it contains two carboxylate functionalities and a cyclohexadiene base structure. Whereas **1** features an arrangement of conjugated double bonds lacking a hydroxyl functionality in position C-4, chorismate possesses cross-conjugated double bonds with a hydroxyl in position C-4. With these alterations, NH_3_ attack and the elimination of H_2_O should be disabled in **1**. In addition, the glycolate side chain in 1 confers additional stability compared with the pyruvate side chain in chorismate. Ligand **2** is similar to **1**, but features an additional amino functionality in position C-6 *anti* to the glycolate side chain. This variation should increase stability as anti-elimination of the side chain is hampered. In addition, **2** bears strong resemblance to ADIC making it a possible product-type inhibitor of PhzE. As an ether derivative of DHHA, ligand **2** should also be a putative PhzD inhibitor that could be resistant to enzymatic ether cleavage. In our synthetic sequence towards ligand **2**, we could show that late-stage derivatization is possible, thus allowing the synthesis of a diverse set of ADIC analogues in the future. In crystallization experiments of PhzE, chorismic acid was found to be converted to benzoate and pyruvate [12]; however, ligands **1** and **2** may remain stable in the active site thus allowing the investigation of binding differences between PhzE and AS.

The synthesis of racemic ligand **1** started with the preparation of the literature-known *rac*-methyl 5-hydroxycyclohexa-1,3-diene-1-carboxylate (**4**), following the method developed by Brion (Scheme 3) [19].

**Figure:**
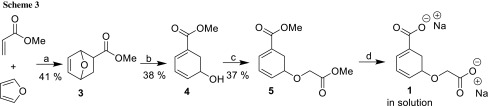

Alcohol **4** was isolated as an oil that degrades slowly upon storage at − 20 °C, as indicated by TLC. To introduce a glycolate side chain in **4**, a Williamson etherification using NaH at low temperature was performed to deliver advanced intermediate *rac*-methyl 5-(2-methoxy-2-oxoethoxy)cyclohexa-1,3-diene-1-carboxylate (**5**) in 37% yield. The planned hydrolysis of the methyl ester moieties in **5** proved to be a considerable synthetic challenge, as **5** is fairly unstable under basic conditions leading to aromatization even upon treatment with 4.2 eq. TMSOK [20], which is considered to be a rather mild reagent. In an effort to avoid aromatization, we used NaOH/H_2_O and tested various co-solvents. The best result was obtained when no co-solvent was used. Gratifyingly, when 2.05 eq. NaOH in H_2_O was used, a clean ^1^H NMR of **1** could be recorded. It has to be noted that possible side products or unreacted starting material could not be detected, but this may be due to their insolubility in D_2_O/H_2_O. Full characterisation of **1** was not performed as aromatization occurred upon concentration. Due to its intrinsic instability **1** will only find limited use as a tool compound.

For the synthesis of racemic ligand **2**, we first followed the literature-known procedure towards advanced intermediate *rac*-ethyl *trans*-6-[(*tert*-butoxycarbonyl)amino]-5-hydroxycyclohexa-1,3-diene-1-carboxylate (**10**) according to Steel and co-workers [21–24], and only minor modifications were made (Scheme 4). Dienophile ethyl (*E*)-3-nitroprop-2-enoate (**7**) was prepared in two steps in a combined yield of 47% [25].

**Figure:**
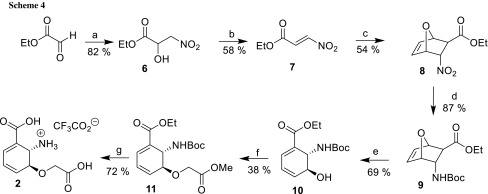

The introduction of the glycolate side chain in **10** using methylbromoacetate as electrophile required substantial optimization of reaction conditions. In our initial attempts, we used Ag_2_O and DBU as bases, but achieved only minor product formation. Various alkali carbonates and additives such as NaI and KI led also to unsatisfactory results. Gratifyingly, when we switched to the stronger base NaH, product yields were significantly improved. At − 10 °C, side product formation could be reduced to a minimum. When pursuing this reaction on a 700 mg scale, we warmed the reaction mixture within 4 h from − 20 to 0 °C and could isolate *rac*-ethyl *trans*-6-[(*tert*-butoxycarbonyl)amino]-5-(2-methoxy-2-oxoethoxy)cyclohexa-1,3-diene-1-carboxylate (**11**) in 38% yield along with 32% of recovered starting material. For completion of the synthesis of inhibitor **2**, the remaining protecting groups had to be removed. The best results were obtained when Boc-removal was performed prior to ester hydrolysis. In the first step, Boc-removal in **11** was achieved using TFA, followed by ester hydrolysis using aqueous KOH in THF. Upon re-acidification with TFA, the desired inhibitor **2** was isolated in 72% yield as a racemic mixture along with CF_3_CO_2_K. Gratifyingly, **2** was found to be a stable ADIC analogue, as no degradation was detected after 5 days in D_2_O, whereas ADIC has a half-life [18] of only approx. 34 h in Tris buffer at pH 8.0.

Potential ligands **1** and **2** will give valuable insights into the binding differences between PhzE and AS. However, it would be advantageous to have access to various additional ligands for a structure–activity relationship (SAR) assessment. For this reason, we exploited the synthesis of potential ligand **2** for the preparation of *rac*-ethyl 5-(2-amino-2-oxoethoxy)-6-[(*tert*-butoxycarbonyl)amino]cyclohexa-1,3-diene-1-carboxylate (**12**) and *rac*-ethyl 6-[(*tert*-butoxycarbonyl)amino]-5-(2-ethoxy-2-oxoethoxy)cyclohex-1-ene-1-carboxylate (**13**) to show that late-stage derivatization is a viable strategy for the synthesis of additional ligand candidates. Advanced intermediates **12** and **13** could be transformed by standard Boc-deprotection into ester prodrugs susceptible to intracellular ester hydrolysis, which should offer advantages over the analogous structures with free carboxylic acids, which might be too charged to cross bacterial cell walls [26].

For the synthesis of **12**, we treated **10** with NaH and iodoacetamide and could isolate ether derivative 12 in 47% yield (Scheme 5).

**Figure:**
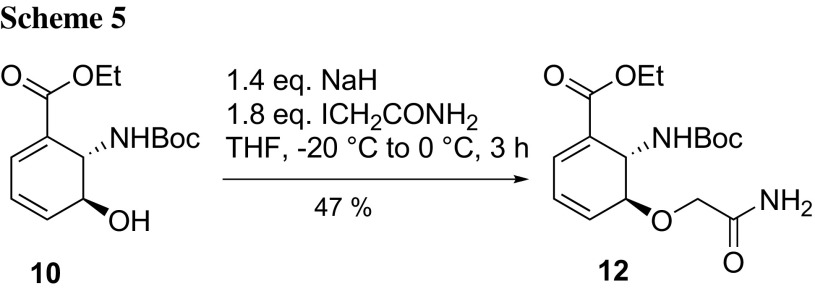

A more advanced probe design led to compound **13**, in which only a single olefin remains, which should make this compound resistant against degradation via aromatization. Hydrogenation of **9** using an H-cube^®^ flow reactor (1 bar H_2_, 10% Pd/C, rt, 1 h) furnished *rac*-ethyl 3-[(*tert*-butoxycarbonyl)amino]-7-oxabicyclo[2.2.1]heptane-2-carboxylate (**14**) [27] in 99% yield without requiring any purification step (Scheme 6). For the subsequent base-mediated opening of bicycle **14**, freshly prepared LiHMDS at − 45 °C led only to unsatisfactory conversion (19% yield of **15** + 65% **14**). With KHMDS and warming from − 45 to − 25 °C, substantial side product formation was observed via TLC. However, at − 50 °C, compound **15** could be obtained in satisfactory 38% yield. For the introduction of the glycolate side chain, we used the neutral conditions of Rh_2_(OAc)_4_-catalyzed OH-insertion with ethyl diazoacetate and were able to isolate **13** in 53% yield.

**Figure:**
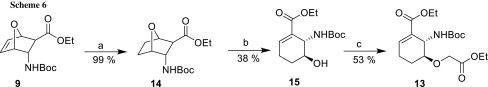

We envisage ligands **1** and **2** to serve as model compounds in the studies of binding differences between PhzE and AS. In addition, inhibitory constants will enable a comparison with known inhibitors of AS [28–30]. Most importantly, we could show that late-stage derivatization at two points in the synthetic protocol towards **2** is possible, which will allow the synthesis of additional ligands for a SAR assessment.

## Conclusion

In summary, we have presented two independent strategies towards ligands **1** and **2**. Both ligands are aimed to interfere with phenazine biosynthesis in bacteria at the earliest possible stage with the overriding goal to develop new antibiotics. Importantly, we could demonstrate that the synthetic strategy towards ligand **2** allows to access various derivatives by branching off at two stages of the synthesis, enabling the study of structure–activity relationship (SAR). Notably, the stable ligand **2** is designed to serve as a dual-inhibitor of both PhzE and PhzD, the first two enzymes in the biocatalytic cascade towards strain-specific phenazines. In the future, the biophysical characterization of these ligands will be performed.

## Experimental

Reactions were carried out under air, unless indicated otherwise. For inert reactions, standard Schlenk techniques under an inert atmosphere of N_2_ or Ar and anhydrous solvents were used. In some cases, different batches of intermediates were pooled and used for a reaction and ^1^H NMR was used to assess conformity. The described nuclear resonance spectra were acquired with the following instruments: Bruker AVANCE III with Autosampler: 300.36 MHz ^1^H NMR, 75.53 MHz ^13^C NMR; Varian Unity Inova: 499.91 MHz ^1^H NMR, 125.69 MHz ^13^C NMR, 470.35 MHz ^19^F NMR; Bruker MSL 300 MHz: 282 MHz ^19^F NMR. Chemical shifts *δ* are referenced to residual protonated solvent signals as internal standard D_2_O: *δ* = 4.79 ppm (^1^H), C_6_D_6_: *δ* = 7.16 ppm (^1^H), 128.06 ppm (^13^C), and CDCl_3_: *δ* = 7.26 ppm (^1^H), 77.16 ppm (^13^C). Signal multiplicities are abbreviated as bs (broad singlet), d (doublet), dd (doublet of doublet), dt (doublet of triplet), m (multiplet), s (singlet), t (triplet), and q (quadruplet). The deuterated solvent, the chemical shifts *δ* in ppm (parts per million), and the coupling constants *J* in Hertz (Hz) are given. Deuterated solvents for nuclear resonance spectroscopy were purchased from Eurisotop^®^ (CDCl_3_, C_6_D_6_) and Deutero^®^ (D_2_O). Analytical thin-layer chromatography (TLC) was performed on Merck silica gel 60-F_254_ plates and spots were visualized by UV-light (*λ* = 254 and/or 366 nm), or by treatment with cerium ammonium molybdate solution (CAM: 2.0 g Ce(IV)SO_4_, 50 g (NH_4_)_2_MoO_4_, 50 cm^3^ concentrated H_2_SO_4_ in 400 cm^3^ water) or KMnO_4_ solution (3.0 g KMnO_4_ and 20.0 g K_2_CO_3_ dissolved in 300 cm^3^ of a 5% NaOH solution). Flash column chromatography was performed using silica gel 60 Å (35–70 µm particle size) from Acros Organics at an air pressure of ~ 1.5 bar. Analytical HPLC measurements were performed on a Shimadzu Nexera Liquid Chromatograph. The separation of the analytes was carried out using a C-18 reversed-phase column of the type “Poroshell^®^ 120 SB-C18, 3.0 × 100 mm, 2.7 μm “by Agilent Technologies, and detection was accomplished with a “Shimadzu SPD-M20A Prominence Diode Array Detector” at a wavelength of *λ* = 210 nm and with the mass selective detector “Shimadzu LCMS-2020 Liquid Chromatograph Mass Spectrometer” in the modes “ESI positive” and “ESI negative”. Two different methods were used: “standard-1”: 0.00–0.50 min 70% water/HCO_2_H and 30% CH_3_CN, 0.50–6.50 min linear to 100% CH_3_CN, 6.50–7.20 min 100% CH_3_CN, 7.20–7.30 min linear to 30% CH_3_CN, 7.30–9.00 min 30% CH_3_CN; 0.7 cm^3^ min^−1^; 40 °C or “standard-2”: 0.00–1.50 min 100% water/HCO_2_H and 0% CH_3_CN, 1.50–5.50 min linear to 80% CH_3_CN, 5.50–6.00 min 80% CH_3_CN, 6.00–6.05 min linear to 100% CH_3_CN, 6.05–6.70 min 100% CH_3_CN, 6.70–6.80 min linear to 0% CH_3_CN, 6.80–8.00 min 0% CH_3_CN; 0.7 cm^3^ min^−1^; 40 °C. High-resolution mass spectrometry (HRMS): TOF MS EI was performed on a Waters GCT premier micromass with an electron impact ionization (EI)-source (70 eV) and samples were injected via an Agilent Technologies GC 7890A with capillary column (DB-5MS, 30 m × 0.25 mm × 0.25 µm film). FT ICR–MS ESI was performed on a LTQ FT Ultrainstrument (Thermo Scientific) with an ESI source and samples were injected using a syringe pump with a flow of 3 mm^3^ min^−1^ (capillary temperature was set to either 200 or to 270 °C). Melting points were determined on a Mel-Temp^®^ melting-point apparatus (Electrothermal). High-pressure hydrogenation experiments were performed using the H-Cube™ continuous hydrogenation unit (HC-2.SS) from Thales Nanotechnology Inc. running with a Knauer Smartline pump 100 and equipped with a 10 cm^3^ ceramic pump head. As hydrogenation catalyst 10% Pd/C catalyst cartridges were used (Thales Nanotechnology inc., THS01111, 10% Pd/C CatCart™). Chemicals were purchased from the companies ABCR, ACROS Organics, Alfa Aesar, Brenntag, Fisher Scientific, Fluka, Merck, Roth, Sigma-Aldrich or VWR and were used without further purification, unless stated otherwise. THF and Et_2_O were distilled, and stored over KOH. For inert reactions, CH_2_Cl_2_ was dried over CaH_2_ and distilled under an argon atmosphere before use. For inert reactions, THF was dried over sodium and distilled under an argon atmosphere before use.

### rac-5-(Carboxylatomethoxy)cyclohexa-1,3-dienecarboxylate disodium salt solution (**1**, C_9_H_8_Na_2_O_5_)

A N_2_-flushed Schlenk flask was charged with 50.0 mg of **5** (0.221 mmol) and 1.04 cm^3^ H_2_O and the mixture was degassed (ultrasound bath) and cooled to 0 °C (ice bath). To the stirred solution, 400 mm^3^ of a NaOH stock solution (1.13 M NaOH in H_2_O) was added and the solution turned slightly yellow. The solution was stirred 12 min at 0 °C and 18 h at rt, after which an NMR sample was taken. ^1^H NMR (300 MHz, CDCl_3_): *δ* = 6.76 (d, *J* = 3.2 Hz, 1H), 6.34 (dd, *J* = 9.5 Hz, 5.6 Hz, 1H), 6.23–6.14 (m, 1H), 4.30–4.19 (m, 2H), 3.88 (s, 2H), 3.34 (s, MeOH), 2.85 (dd, *J* = 19.0 Hz, 4.4 Hz, 1H), 2.53 (dd, *J* = 18.9 Hz, 7.7 Hz, 1H) ppm. Side products or unreacted starting material was not detected and this is maybe due to insolubility in D_2_O/H_2_O. Full characterisation of **1** was not performed as aromatization occurred upon concentration.

### rac-trans-2-Carboxy-6-(carboxymethoxy)cyclohexa-2,4-dien-1-ammonium 2,2,2-trifluoroacetate (**2**, C_11_H_12_F_3_NO_7_)

In a one-neck round-bottom flask, 7.5 cm^3^ trifluoroacetic acid (98 mmol) was added dropwise within 5 min to a stirred solution of 149 mg of **11** (418 µmol) in 7.5 cm^3^ CH_2_Cl_2_. After 2 h, complete consumption of the starting material was observed (reaction monitoring via HPLC) and the pale yellow solution was concentrated in vacuo. The resulting solid was dissolved in 4 cm^3^ THF and treated with 2.89 cm^3^ 1.74 M KOH solution (5.02 mmol). The mixture was stirred at rt until complete consumption of the starting material was observed (reaction monitoring via HPLC). Subsequently, the solution was diluted with 3 cm^3^ H_2_O and washed with Et_2_O (2 × 2 cm^3^). The aqueous layer was then acidified with 50% trifluoroacetic acid to pH 2 and concentrated in vacuo to afford a mixture of 2 and potassium trifluoroacetate. The mass concentration of compound **2** was determined via ^1^H NMR-spectroscopy using trimethylamine hydrochloride (*δ*(D_2_O) = 2.90 ppm) as an external standard: in an NMR tube 100 mm^3^ of a 1.81 g dm^−3^ Me_3_N·HCl solution in D_2_O was added to a solution of 23.4 mg of the obtained mixture in D_2_O using a 250 mm^3^ Hamilton syringe. The mass concentration was calculated via integration. Yield: 738 mg (13 wt% mixture with F_3_CCO_2_K, 72%), off-white solid. HPLC–MS (“standard-2”): *t*_*R*_ = 2.45 min (*m/z* = 214 ([M + H]^+^), *λ*_max_ = 280 nm); ^1^H NMR (500 MHz, D_2_O): *δ* = 7.35 (d, *J* = 5.4 Hz, 1H), 6.52–6.42 (m, 2H), 4.57 (d, *J* = 5.7 Hz, 1H), 4.51–4.46 (m, 1H), 4.27 (d, *J* = 16.6 Hz, 1H), 4.22 (d, *J* = 16.7 Hz, 1H) ppm; The relative stereochemistry was determined via ^1^H NMR via a coupling constant comparison with ADIC [31]; ^13^C NMR (126 MHz, D_2_O): *δ* = 175.3, 169.1, 162.9 (q, *J* = 35.5 Hz), 136.3, 129.4, 126.2, 123.9, 116.3 (q, *J* = 291.7 Hz), 73.5, 66.1, 48.2 ppm; ^19^F NMR (470 MHz, D_2_O): *δ* = − 75.6 (F_3_CCO_2_^−^) ppm; HRMS (FTICR MS ESI): *m/z* calcd for [M + H]^+^ 214.0710, found 214.0711.

### rac-Methyl 7-oxabicyclo[2.2.1]hept-5-ene-2-carboxylate (**3**)

The synthesis was performed in analogy to Ref. [19]. A flame-dried and argon-flushed Schlenk flask was charged with 5.10 g ZnI_2_ (16.0 mmol), 5.60 cm^3^ furan (77.3 mmol), and 4.78 cm^3^ methyl acrylate (52.8 mmol). The mixture was stirred at 40 °C for 3 days, after which 150 cm^3^ EtOAc was added. The organic layer was washed with 1 M Na_2_S_2_O_3_ solution (1 × 80 cm^3^), dried over Na_2_SO_4_, filtered, and concentrated in vacuo. The crude product was purified via silica gel filtration (cyclohexane to cyclohexane/EtOAc = 1/1) and 4.88 g (41%) of **3** was isolated as slightly yellow liquid. *R*_*f*_ = 0.30 (cyclohexane/EtOAc = 4/1, CAM); ^1^H NMR (300 MHz, CDCl_3_; *endo*/*exo*-mixture): *δ* = 6.49–6.13 (m, 2H), 5.21–5.09 (m, 1H), 5.03 (m, 1H), 3.67 (2 s, 3H), 3.17–3.00 (m, 0.3H), 2.42 (m, 0.7 H), 2.13 (m, 1H), 1.65–1.43 (m, 1H) ppm; ^13^C NMR (APT, 76 MHz, CDCl_3_; *endo*/*exo*-mixture): *δ* = 174.3, 172.7, 137.2, 134.8, 132.7, 81.0, 79.1, 78.8, 78.1, 52.2, 51.8, 42.8, 42.8, 29.2, 28.6 ppm.

### rac-Methyl 5-hydroxycyclohexa-1,3-diene-1-carboxylate (**4**)

The synthesis was performed in analogy to Ref. [19]. A flame-dried and argon-flushed two-neck flask was charged with 1.28 cm^3^ hexamethyldisilazane (6.14 mmol) and 35 cm^3^ anhydrous THF. The mixture was stirred and cooled to − 78 °C (dry ice/acetone) and 2.58 cm^3^ 2.21 M *n*-BuLi (5.70 mmol) were slowly added. Subsequently, the mixture was warmed to 0 °C and held at that temperature for 15 min, after which the mixture was again cooled to − 78 °C. Subsequently, 790 mg of **3** (5.12 mmol) dissolved in 6.5 cm^3^ anhydrous THF was added over the course of 10 min, after which the mixture was slowly warmed to − 47 °C in the cooling bath. After 120 min, the solution was poured into 200 cm^3^ saturated NH_4_Cl solution (pre-cooled to 0 °C). The mixture was extracted with CH_2_Cl_2_ (4 × 50 cm^3^) and the combined organic layers were dried over Na_2_SO_4_, filtered, and concentrated in vacuo. The crude product was purified via flash column chromatography (cyclohexane/EtOAc = 3/1) and 309 mg (38%) of **4** was isolated as colorless oil. *R*_*f*_ = 0.50 (cyclohexane/EtOAc = 1/1, UV); ^1^H NMR (300 MHz, CDCl_3_): *δ* = 7.14–7.01 (m, 1H), 6.30–6.17 (m, 2H), 4.37 (m, 1H), 3.77 (s, 3H), 2.99–2.84 (ddd, *J* = 18.8 Hz, 5.1 Hz, 0.8 Hz, 1H), 2.70–2.56 (ddd, *J* = 18.9 Hz, 7.6 Hz, 2.3 Hz, 1H), 1.74 (s, 1H) ppm; ^13^C NMR (APT, 76 MHz, CDCl_3_): *δ* = 167.6, 133.4, 131.6, 127.1, 124.9, 63.3, 52.0, 31.3 ppm.

### rac-Methyl 5-(2-methoxy-2-oxoethoxy)cyclohexa-1,3-diene-1-carboxylate (**5**, C_11_H_14_O_5_)

A flame-dried and argon-flushed Schlenk flask was charged with 612 mg of **10** (3.97 mmol) and 20 cm^3^ anhydrous THF. The mixture was cooled to − 78 °C (dry ice/acetone) and 463 mg NaH (11.6 mmol, 60% dispersion in mineral oil) was slowly added. The mixture was stirred at − 78 °C for 30 min, then at − 38 °C for 15 min, after which 800 mm^3^ (8.47 mmol) methylbromoacetate was added. Within 60 min, the reaction mixture was slowly warmed to − 21 °C in the cooling bath, after which complete consumption of the starting material was observed (reaction monitoring via TLC) and the solution was poured into 300 cm^3^ saturated NH_4_Cl solution (pre-cooled to − 15 °C). The mixture was extracted with EtOAc (4 × 60 cm^3^) and the combined organic layers were dried over Na_2_SO_4_, filtered, and concentrated in vacuo. The crude product was purified via flash column chromatography (cyclohexane/EtOAc = 5/1) and 329 mg (37%) of **5** was isolated as colorless oil; *R*_*f*_ = 0.30 (cyclohexane/EtOAc = 3/1, CAM); ^1^H NMR (300 MHz, CDCl_3_): *δ* = 7.12–7.02 (m, 1H), 6.27 (m, 2H), 4.29 (m, 1H), 4.15–3.99 (m, 2H), 3.77, 3.74 (2 s, 6H), 3.04–2.90 (m, 1H), 2.60 (m, 1H) ppm; ^13^C NMR (APT, 76 MHz, CDCl_3_): *δ* = 171.0, 167.3, 131.6, 130.1, 127.5, 126.5, 70.9, 64.9, 52.0, 51.9, 27.8 ppm; HRMS (TOF MS EI): *m/z* calcd for [M]^+^ 226.0841, found 226.0846.

### Ethyl 2-hydroxy-3-nitropropanoate (**6**)

The synthesis was performed in analogy to Ref. [25]. A two-neck round-bottom flask equipped with a drying tube (CaCl_2_) was charged with 54 cm^3^ ethyl glyoxylate solution (0.27 mol, ca. 50% solution in toluene), 126 cm^3^ nitromethane (2.33 mol), and 54.0 g aluminum oxide (0.53 mol, activated, neutral). The mixture was stirred and heated at reflux temperature for 48 h until complete consumption of the starting material was observed (reaction monitoring via TLC). The resulting brownish-red suspension was allowed to cool to rt, filtered through a glass frit and the filter cake was washed with EtOAc (3 × 100 cm^3^). The solvent was removed in vacuo to furnish a brownish-red oil, which was purified via flash column chromatography (cyclohexane/EtOAc = 2/1 to cyclohexane/EtOAc = 1/2). The resulting brownish-red oil was crystallized in the fridge overnight and 35.4 g (82%) of **6** was isolated as orange to pale yellow crystals. *R*_*f*_ = 0.26 (cyclohexane/EtOAc = 2/1, CAM); m.p.: 35–37 °C; ^1^H NMR (300 MHz, CDCl_3_): *δ* = 4.77 (d, *J* = 4.1 Hz, 2H), 4.63 (m, 1H), 4.40–4.30 (m, 2H), 3.33 (d, *J* = 4.1 Hz, 1H), 1.33 (t, *J* = 7.1 Hz, 3H) ppm; ^13^C NMR (APT, 76 MHz, CDCl_3_): *δ* = 170.8, 76.9, 67.7, 63.3, 14.2 ppm.

### Ethyl (E)-3-nitroprop-2-enoate (**7**)

The synthesis was performed in analogy to Ref. [25]. A flame-dried and argon-flushed 1000 cm^3^ three-neck round-bottom flask equipped with a pressure-equalized dropping funnel and a gas bubbler was charged with 27.2 g of **6** (167 mmol) and 340 cm^3^ anhydrous CH_2_Cl_2_. The solution was cooled to − 20 °C (dry ice/acetone) and 39 cm^3^ methanesulfonyl chloride (504 mmol) was added dropwise within 20 min. Subsequently, 71 cm^3^ anhydrous triethylamine (509 mmol) was added dropwise within 30 min, whereupon a reddish-brown suspension was formed. The mixture was allowed to warm to rt and was stirred overnight. After 17 h, complete consumption of the starting material was observed (reaction monitoring via TLC). The suspension was poured into 1200 cm^3^ ice-cold H_2_O and stirred for 10 min. The organic layer was separated and the aqueous layer was extracted with CH_2_Cl_2_ (2x300 cm^3^). The combined organic layers were washed with H_2_O (3 × 200 cm^3^), dried over Na_2_SO_4_, filtered, and concentrated in vacuo to furnish a brownish oil. The crude product was purified via fractional distillation (14 cm Vigreux column, 0.72 mbar, 36–50 °C) to furnish impure fractions of product containing different amounts of methanesulfonyl chloride. Subsequently, a flash column chromatography (cyclohexane/EtOAc = 19/1) was performed and 14.0 g (58%) of **7** was isolated as bright yellow oil with pungent odor. *R*_*f*_ = 0.62 (cyclohexane/EtOAc = 5/1, KMnO_4_); b.p.: 48–50 °C (0.70 mbar); ^1^H NMR (300 MHz, CDCl_3_): *δ* = 7.67 (d, *J* = 13.5 Hz, 1H), 7.08 (d, *J* = 13.5 Hz, 1H), 4.32 (q, *J* = 7.1 Hz, 2H), 1.34 (t, *J* = 7.1 Hz, 3H) ppm; ^13^C NMR (APT, 76 MHz, CDCl_3_): *δ* = 162.8, 149.1, 127.8, 62.6, 14.1 ppm.

### rac-Ethyl endo-3-nitro-7-oxabicyclo[2.2.1]hepta-5-ene-exo-2-carboxylate (**8**)

The synthesis was performed in analogy to Ref. [22]. In a one-neck round-bottom flask 23.9 cm^3^ furan (329 mmol) was added to a stirred and cooled (− 20 °C, acetone bath/cryostat) solution of 23.8 g (164 mmol) of **7** in 90 cm^3^ chloroform. The flask was covered with aluminum foil to exclude light and the reaction mixture was stirred at − 20 °C for 5 d and then at rt for 2 days (reaction monitoring via TLC). Subsequently, the orange solution was concentrated in vacuo to furnish a yellow solid. The crude product was purified via flash column chromatography (cyclohexane/EtOAc = 8/1). Fractions containing both diastereomers were purified via two additional flash column chromatographies (cyclohexane/EtOAc = 8/1) and 18.8 g (54%) of **8** was isolated as colorless crystals. *R*_*f*_ = 0.40 (cyclohexane/EtOAc = 3/1, CAM); m.p.: 49–50 °C; ^1^H NMR (300 MHz, C_6_D_6_): *δ* = 5.76 (m, 2H), 5.34 (dd, *J* = 4.8 Hz, 3.1 Hz, 1H), 4.91 (s, 1H), 4.85 (d, *J* = 4.7 Hz, 1H), 3.84 (q, *J* = 7.1 Hz, 2H), 2.93 (d, *J* = 3.0 Hz, 1H), 0.85 (t, *J* = 7.1 Hz, 3H) ppm; ^13^C NMR (APT, 76 MHz, C_6_D_6_): *δ* = 169.6, 138.7, 133.7, 84.7, 83.3, 79.1, 61.7, 49.3, 14.0 ppm.

### rac-Ethyl endo-3-[(tert-butoxycarbonyl)amino]-7-oxabicyclo[2.2.1]hept-5-ene-exo-2-carboxylate (**9**)

The synthesis was performed in analogy to Ref. [22]. A one-neck round-bottom flask equipped with a gas bubbler was charged with 18.2 g oxanorbornene** 8** (85.4 mmol) and 790 cm^3^ EtOH. The yellow solution was cooled to 0 °C (ice bath) and 120 cm^3^ concentrated HCl (1.44 mol) was added, followed by the portion-wise addition of 110.8 g activated zinc dust (1.69 mol; activation by washing twice with 1 M HCl, H_2_O, MeOH and subsequent drying in vacuo). The gray suspension was stirred at 0 °C for 30 min and then at rt for 18 h (reaction monitoring via TLC). Subsequently, the suspension was filtered through a pad of Celite and the filter cake was washed with EtOH (1 × 300 cm^3^). The filtrate was then transferred into a 2000 cm^3^ one-neck round-bottom flask equipped with a pressure-equalizing dropping funnel and a gas bubbler, after which 195 cm^3^ triethylamine (1.40 mol) was added dropwise. The resulting colorless suspension was treated with 51.0 g di-*tert*-butyl dicarbonate (234 mmol) and stirred at rt for 24 h (reaction monitoring via TLC). Subsequently, the reaction mixture was concentrated in vacuo to furnish a pale yellow solid, which was dissolved in EtOAc (1 × 1400 cm^3^). The organic layer was washed with H_2_O (1 × 1100 cm^3^) and the aqueous layer was then back-extracted with EtOAc (1 × 200 cm^3^). The combined organic layers were washed with saturated NaHCO_3_ solution (1x450 cm^3^), dried over Na_2_SO_4_, filtered, and concentrated in vacuo. The crude product was purified via flash column chromatography (cyclohexane/EtOAc = 3/1 to EtOAc) and 21.1 g (87%) was isolated as colorless solid. *R*_*f*_ = 0.29 (cyclohexane/EtOAc = 2/1, CAM); m.p.: 101 °C; ^1^H NMR (300 MHz, CDCl_3_): *δ* = 6.61 (dd, *J* = 5.8 Hz, 1.6 Hz, 1H), 6.47 (dd, *J* = 5.8 Hz, 1.4 Hz, 1H), 5.13 (s, 1H), 5.07 (bs, 1H), 4.55 (bs, 1H), 4.21 (q, *J* = 7.1 Hz, 3H), 2.05 (d, *J* = 3.5 Hz, 1H), 1.44 (s, 9H), 1.29 (t, *J* = 7.1 Hz, 3H) ppm; ^13^C NMR (APT, 76 MHz, CDCl_3_): *δ* = 171.9, 155.1, 138.0, 134.6, 82.2, 79.1, 61.4, 53.3, 52.6, 28.5, 14.3 ppm (one ^13^C-signal could not be observed).

### rac-Ethyl trans-6-[(tert-butoxycarbonyl)amino]-5-hydroxycyclohexa-1,3-diene-1-carboxylate (**10**)

The synthesis was performed in analogy to Ref. [21]. A flame-dried and argon-flushed two-neck round-bottom flask was charged with 4.22 g KHMDS (21.2 mmol) and 68 cm^3^ anhydrous THF. The solution was cooled to − 45 °C (dry ice/acetone) and 1.99 g oxanorbornene **9** (7.01 mmol) dissolved in 30 cm^3^ anhydrous THF (pre-cooled to − 45 °C in a dry ice/acetone bath) was added via cannula. The mixture was stirred at − 45 °C for 100 min until complete consumption of the starting material was observed (reaction monitoring via TLC). Subsequently, the reaction mixture was poured into a separatory funnel containing 200 cm^3^ saturated NH_4_Cl solution. The aqueous layer was extracted with EtOAc (3 × 90 cm^3^). The combined organic layers were dried over Na_2_SO_4_, filtered, and concentrated in vacuo to furnish a brownish-yellow oil. The crude product was purified via flash column chromatography (cyclohexane/EtOAc = 3/2 to cyclohexane/EtOAc = 1/1) and 1.37 g (69%) of **10** was isolated as colorless solid. *R*_*f*_ = 0.36 (cyclohexane/EtOAc = 2/3, CAM); m.p.: 96–97 °C; ^1^H NMR (300 MHz, CDCl_3_): *δ* = 7.17 (d, *J* = 4.3 Hz, 1H), 6.27 (m, *J* = 4.2 Hz, 2H), 4.78 (d, *J* = 7.7 Hz, 1H), 4.59–4.12 (m, 5H), 2.63 (bs, 1H), 1.44 (s, 9H), 1.30 (t, *J* = 7.1 Hz, 3H) ppm; ^13^C NMR (APT, 76 MHz, CDCl_3_): 155.6, 133.6, 132.8, 124.7, 68.1, 61.0, 50.4, 28.5, 14.3 ppm (^13^C-signals of quaternary carbon atoms could not be observed).

### rac-Ethyl trans-6-[(tert-butoxycarbonyl)amino]-5-(2-methoxy-2-oxoethoxy)cyclohexa-1,3-diene-1-carboxylate (**11**, C_17_H_25_NO_7_)

A flame-dried and argon-flushed Schlenk flask was charged with 705 mg of **10** (2.49 mmol), 32 cm^3^ anhydrous THF and the mixture was cooled to − 20 °C (dry ice/acetone). To the stirred, slightly yellow solution 131 mg NaH (3.28 mmol, 60% dispersion in mineral oil) was added, whereupon the solution became cloudy. After 15 min, 415 mm^3^ methyl bromoacetate (4.38 mmol) was added dropwise to the yellow solution, while the temperature was kept at − 20 °C. The mixture was stirred and allowed to warm to 0 °C within 4 h (reaction monitoring via TLC). Subsequently, the reaction mixture was poured into a flask containing 50 cm^3^ ice-cold saturated NH_4_Cl solution. The aqueous layer was extracted with EtOAc (3 × 120 cm^3^). The combined organic layers were dried over Na_2_SO_4_, filtered, and concentrated in vacuo to furnish a yellow oil. The crude product was purified via flash column chromatography (cyclohexane/EtOAc = 4/1 to cyclohexane/EtOAc = 1/2) and 337 mg (38%) of **11** was isolated as colorless solid. In addition, 240 mg of starting material **3** was recovered. *R*_*f*_ = 0.28 (cyclohexane/EtOAc = 2/1, CAM); m.p.: 70–71 °C; ^1^H NMR (300 MHz, CDCl_3_): *δ* = 7.18 (m, 1H), 6.32 (m, 2H), 4.84 (d, *J* = 7.3 Hz, 1H), 4.47–4.13 (m, 5H), 4.08 (s, 1H), 3.74 (s, 3H), 1.42 (s, 9H), 1.29 (t, *J* = 7.1 Hz, 3H) ppm; ^13^C NMR (76 MHz, CDCl_3_): *δ* = 171.2, 165.8, 155.1, 133.7, 130.0, 127.6, 125.8, 80.0, 75.6, 66.2, 61.0, 52.0, 46.2, 28.5, 14.3 ppm (2 peaks are missing); HRMS (TOF MS EI): *m/z* calcd for [M]^+^ 355.1626, found 355.1637.

### rac-Ethyl 5-(2-amino-2-oxoethoxy)-6-[(tert-butoxycarbonyl)amino]cyclohexa-1,3-diene-1-carboxylate (**12**, C_16_H_24_N_2_O_6_)

A flame-dried and argon-flushed Schlenk flask was charged with 250 mg of **10** (0.886 mmol) and 11 cm^3^ anhydrous THF and the mixture was cooled to − 20 °C (dry ice/acetone). To the stirred, slightly yellowish solution 48 mg NaH (1.20 mmol, 60% dispersion in mineral oil) was added, whereupon the solution became cloudy. After 15 min, 291 mg iodoacetamide (1.57 mmol) was added to the yellow solution, while the temperature was kept at − 20 °C. The mixture was stirred and allowed to warm up to 0 °C within 2 h (reaction monitoring via TLC). The mixture was stirred for another 30 min at this temperature. Subsequently, the reaction was poured into 30 cm^3^ saturated NH_4_Cl solution and the aqueous layer was extracted with EtOAc (6 × 30 cm^3^). The combined organic layers were dried over Na_2_SO_4_, filtered, and concentrated in vacuo to furnish a sticky yellow gum. The crude product was purified via flash column chromatography (cyclohexane/EtOAc = 1/4) and 141 mg (47%) of **13** was isolated as yellowish sticky gum. In addition, 47 mg of the starting material was recovered. *R*_*f*_ = 0.28 (cyclohexane/EtOAc = 1/4, CAM); ^1^H NMR (300 MHz, CDCl_3_): *δ* = 7.18 (d, *J* = 5.5 Hz, 1H), 6.54 (bs, 1H), 6.36 (m, 1H), 6.32–6.19 (m, 1H), 5.73 (bs, 1H), 4.85 (d, *J* = 6.9 Hz, 1H), 4.42–4.00 (m, 6H), 1.42 (s, 9H), 1.30 (t, *J* = 7.1 Hz, 3H) ppm. The relative stereochemistry was determined via ^1^H NMR via a coupling constant comparison with compounds 11 and 2 that have been compared to ADIC [31]; ^13^C NMR (APT, 76 MHz, CDCl_3_): *δ* = 172.4, 165.6, 155.2, 133.3, 129.1, 128.1, 126.8, 74.9, 67.7, 61.2, 47.1, 28.5, 14.3 ppm (3 peaks are missing); HRMS (TOF MS EI): *m/z* calcd for [M]^+^ 340.1634, found 340.1647.

### rac-Ethyl 6-[(tert-butoxycarbonyl)amino]-5-(2-ethoxy-2-oxoethoxy)cyclohex-1-ene-1-carboxylate (**13**)

A flame-dried and argon-flushed Schlenk flask was charged with 102.6 mg of **15** (0.360 mmol), 1.0 cm^3^ absolute CH_2_Cl_2_ and 2.6 mg Rh_2_(OAc)_4_ (5.89 µmol). To the stirred solution 64 mg ethyl diazoacetate (0.557 mmol; caution: toxic and explosive), dissolved in 3 cm^3^ absolute CH_2_Cl_2_, was added over 1 h at rt. The solution was stirred for another 60 min, after which full consumption of the starting material was observed (reaction monitoring via TLC). The solution was concentrated in vacuo to furnish a yellow oil. The crude product was purified via flash column chromatography (cyclohexane/EtOAc = 3/1) and 70.4 mg (53%) of **13** was isolated as colorless solid. *R*_*f*_ = 0.20 (cyclohexane/EtOAc = 3/1, CAM); m.p.: 106–107 °C; ^1^H NMR (300 MHz, CDCl_3_): *δ* = 7.22 (s, 1H), 4.51 (d, 1H, *J* = 6.0 Hz), 4.25–4.10 (m, 6H), 3.77 (s, 1H), 2.44 (m, 1H), 2.19 (dt, *J* = 20.2 Hz, 5.0 Hz, 1H), 2.03 (m, 1H), 1.66 (d, *J* = 17.6 Hz, 1H), 1.44 (s, 9 H), 1.27 (2 t, *J* = 7.1 Hz, 6H) ppm. The relative stereochemistry was determined via ^1^H NMR via a coupling constant comparison with compounds **2** and **11** that have been compared with ADIC [31]; ^13^C NMR (76 MHz, CDCl_3_): *δ* = 170.9, 166.2, 154.9, 144.1, 127.5, 79.8, 77.4, 67.0, 60.9, 60.8, 46.0, 28.5, 21.6, 21.5, 14.3 (2 C) ppm (two peaks are missing); HRMS (TOF MS EI^+^): *m/z* calcd for [M-C_4_H_9_ + H]^+^ 315.1313, found 315.1312.

### rac-Ethyl 3-[(tert-butoxycarbonyl)amino]-7-oxabicyclo[2.2.1]heptane-2-carboxylate (**14**)

The synthesis was performed in analogy to Ref. [27]. A colorless 5 mM solution of the substrate was prepared by dissolving 999.2 mg of **9** (3.53 mmol) in 70 cm^3^ MeOH and transferred into a 150 cm^3^ beaker. For the reduction itself a continuous-flow hydrogenation reactor (H-cube^®^) with a 10% Pd/C catalyst cartridge was used with the following conditions: 1.0 cm^3^ min^−1^, rt, atmospheric pressure, full H_2_. The reaction was stopped after multiple runs (ca. 6 times, 8 h). The product solution was transferred into a 250 cm^3^ round-bottom flask and the solvent was removed in vacuo and 995 mg (99%) of **14** was isolated as colorless solid. *R*_*f*_ = 0.73 (cyclohexane/EtOAc = 2/3, KMnO_4_); m.p.: 113–115 °C; ^1^H NMR (300 MHz, CDCl_3_): *δ* = 4.72 (m, 2H), 4.25 (d, *J* = 4.9 Hz, 1H), 4.18 (q, *J* = 7.1 Hz, 2H), 2.15 (d, *J* = 4.7 Hz, 1H), 1.92–1.75 (m, 2H), 1.75–1.47 (m, 3H), 1.43 (s, 9H), 1.26 (t, *J* = 7.1 Hz, 3H) ppm; ^13^C NMR (76 MHz, CDCl_3_): *δ* = 172.2, 155.6, 79.8, 78.5, 77.4, 61.3, 56.2, 55.2, 30.2, 28.4, 22.5, 14.3 ppm (2 peaks are missing).

### rac-Ethyl 6-[(tert-butoxycarbonyl)amino]-5-hydroxycyclohex-1-ene-1-carboxylate (**15**, C_14_H_23_NO_7_)

A flame-dried and argon-flushed Schlenk flask was charged with 12 cm^3^ of absolute THF and 835 mg KHMDS (4.19 mmol). The solution was cooled to − 50 °C (dry ice/acetone). Subsequently, a solution of 403.5 mg of **14** (1.41 mmol) in 6 cm^3^ absolute THF was added. The reaction was stirred for 5 h, when complete consumption of the starting material was observed (reaction monitoring via TLC). The solution was poured into 40 cm^3^ saturated NH_4_Cl solution and the aqueous layer was extracted with EtOAc (3 × 40 cm^3^). The combined organic layers were dried over Na_2_SO_4_, filtered, and concentrated in vacuo. The crude product was purified via flash column chromatography (cyclohexane/EtOAc = 10/1 to cyclohexane/EtOAc = 2/1) and 151 mg (38%) of **15** was isolated as colorless solid. *R*_*f*_ = 0.31 (cyclohexane/EtOAc = 1/1, KMnO_4_); m.p.: 110–112 °C; ^1^H NMR (300 MHz, CDCl_3_): *δ* = 7.18 (s, 1H), 4.53 (s, 1H), 4.38 (s, 1H), 4.31–4.10 (m, 2H), 4.05 (s, 1H), 2.62 (s, 1H), 2.51–2.31 (m, 1H), 2.30–2.13 (m, 1H), 2.13–2.11 (m, 1H), 1.89–1.63 (m, 3H), 1.44 (s, 9H), 1.27 (t, *J* = 7.1 Hz, 3.5H) ppm; ^13^C NMR (APT, 76 MHz, CDCl_3_): *δ* = 166.3, 143.7, 128.0, 69.2, 60.8, 51.0, 28.5, 24.2, 21.8, 14.3 ppm (4 peaks are missing); HRMS (TOF MS EI): *m/z* calcd for [M]^+^ 285.1571, found 285.1574.

## Electronic supplementary material

Below is the link to the electronic supplementary material.

Supplementary material 1 (PDF 1853 kb)
