# Supplementary material for: Synthesis of novel ligands targeting phenazine biosynthesis proteins as a strategy for antibiotic intervention
Source: Monatsh Chem. 2017 Nov 30;149(4):847–56. doi: 10.1007/s00706-017-2100-z (PMC5906492; doi:10.1007/s00706-017-2100-z)
Supplement: Supplementary file 1 — Supplementary material 1 (PDF 1853 kb) [file 706_2017_2100_MOESM1_ESM.pdf]

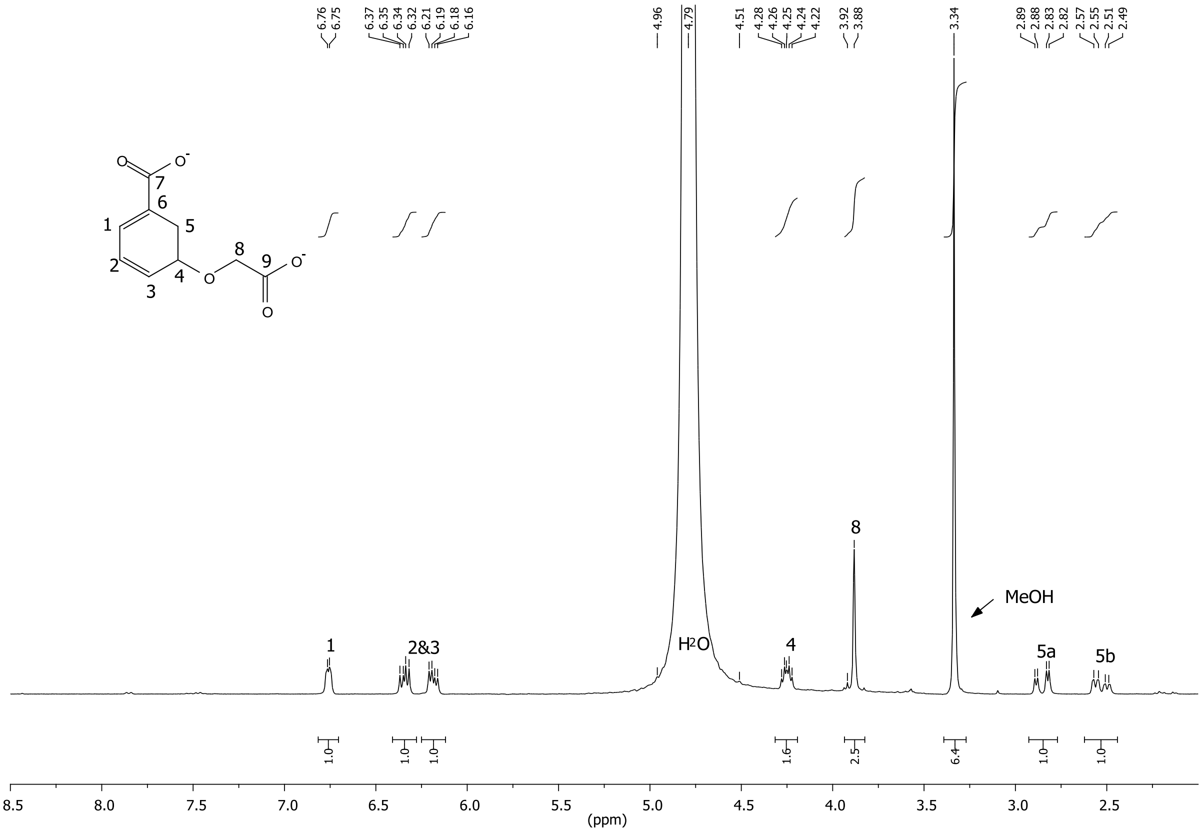

^{1}H-NMR (300 MHz, D_2O) of hydrolysed **5** (= compound **1**) without workup in H_2O

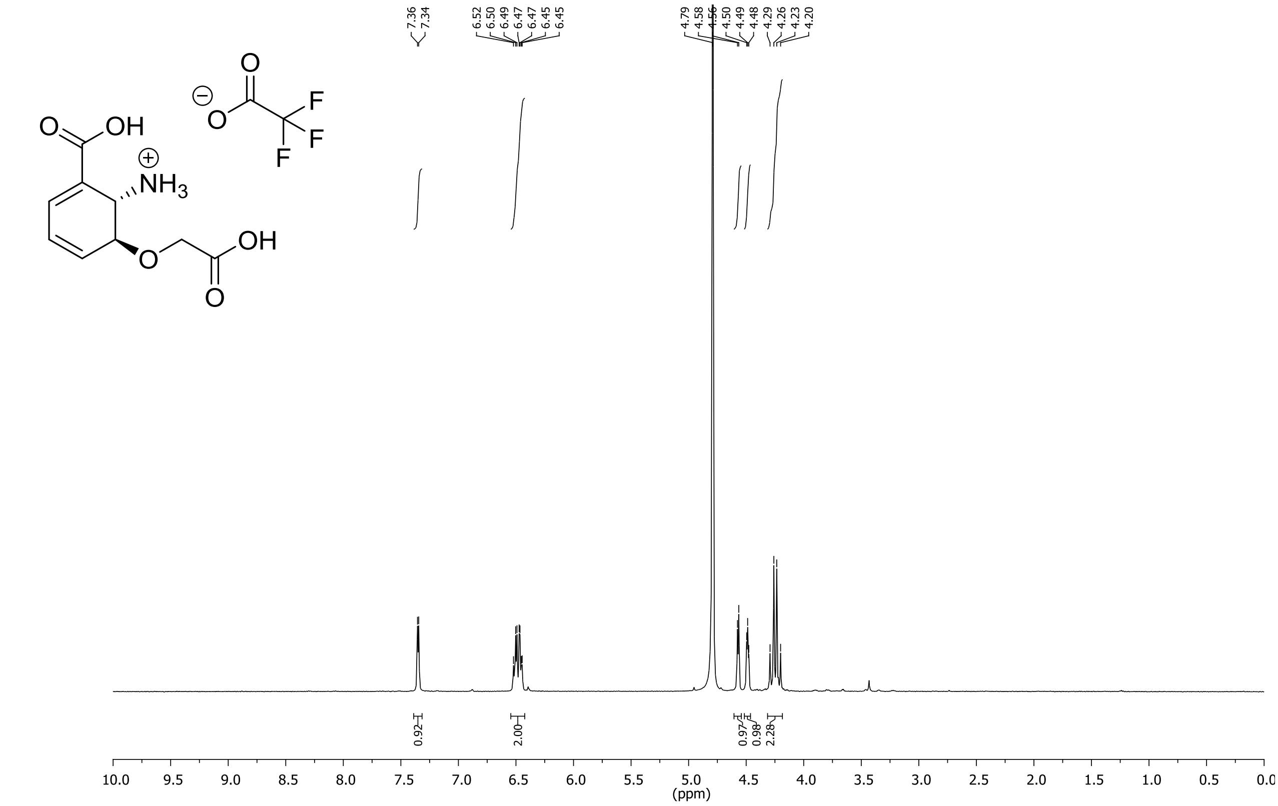

^{1}H-NMR (499.88 MHz, D_2O) of compound **2**

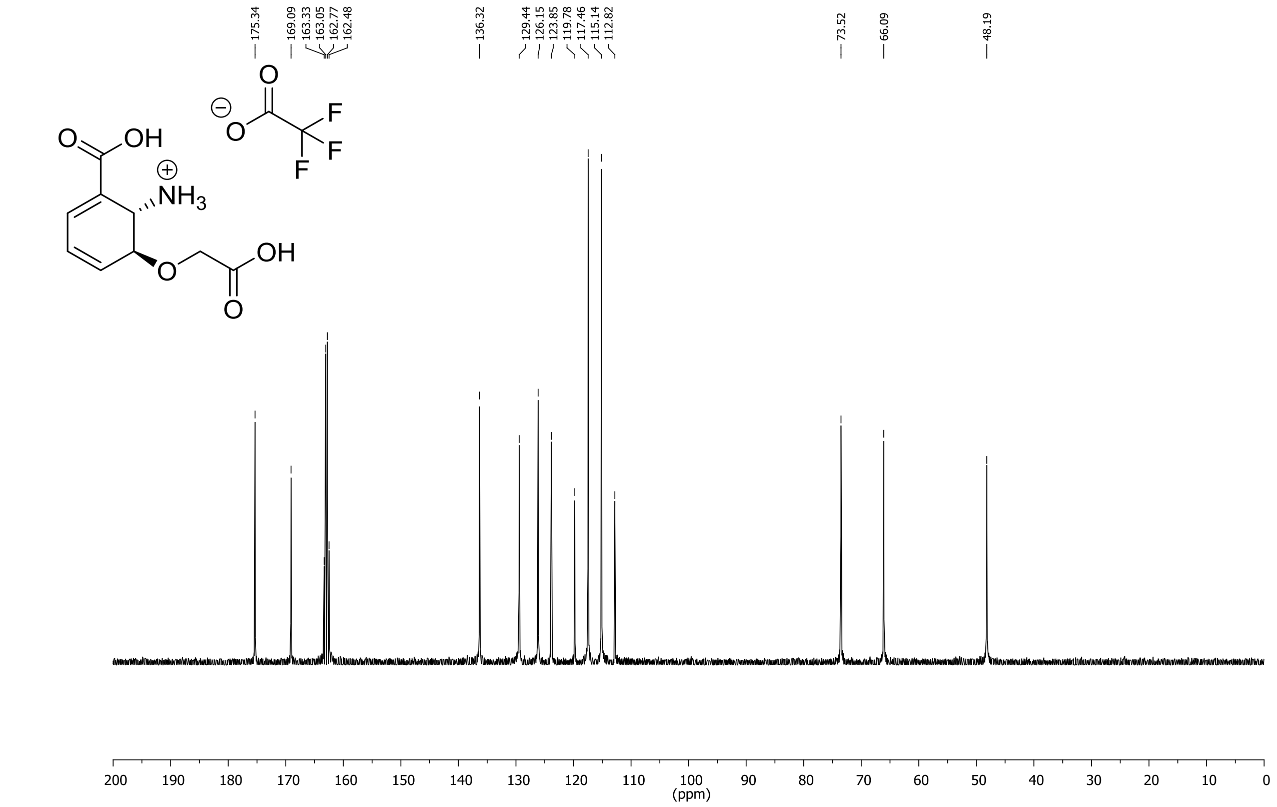

^{13}C-NMR (125.69 MHz, D_2O) of compound **2**

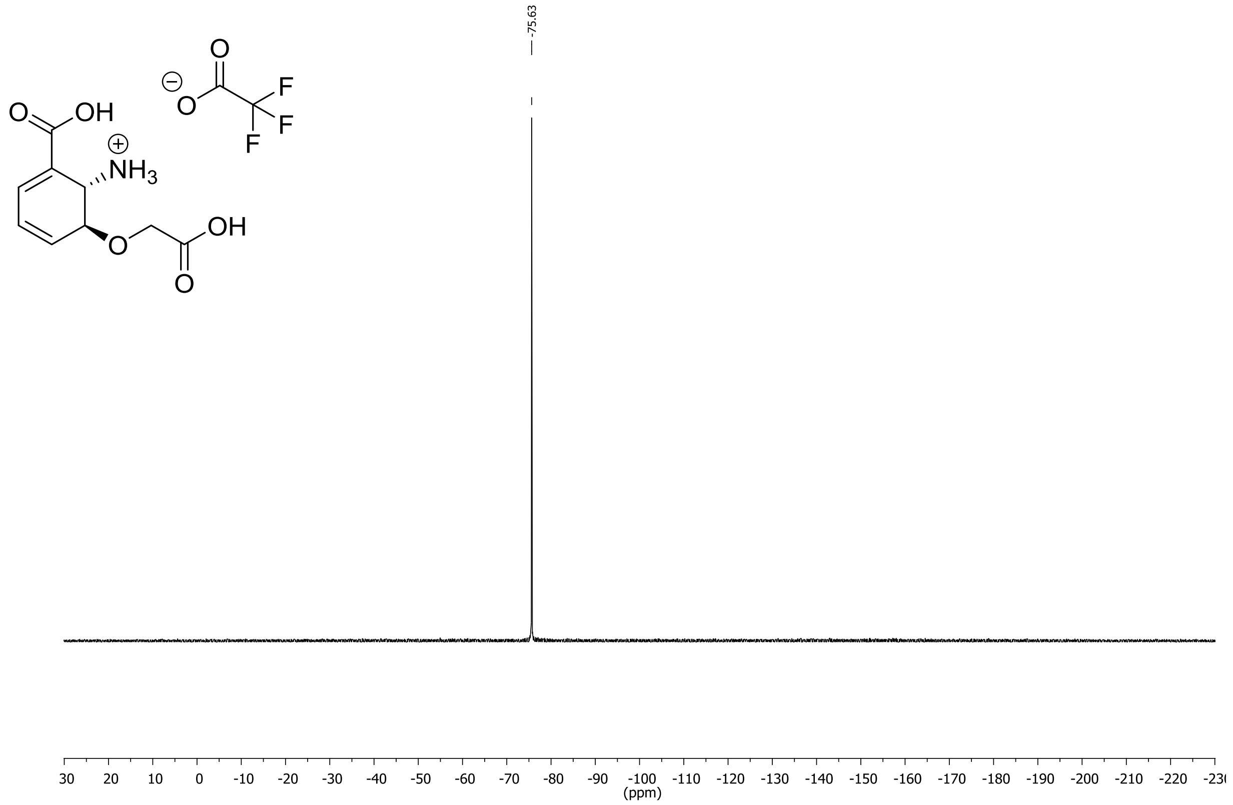

^{19}F-NMR (470.35 MHz, D_2O) of compound **2**

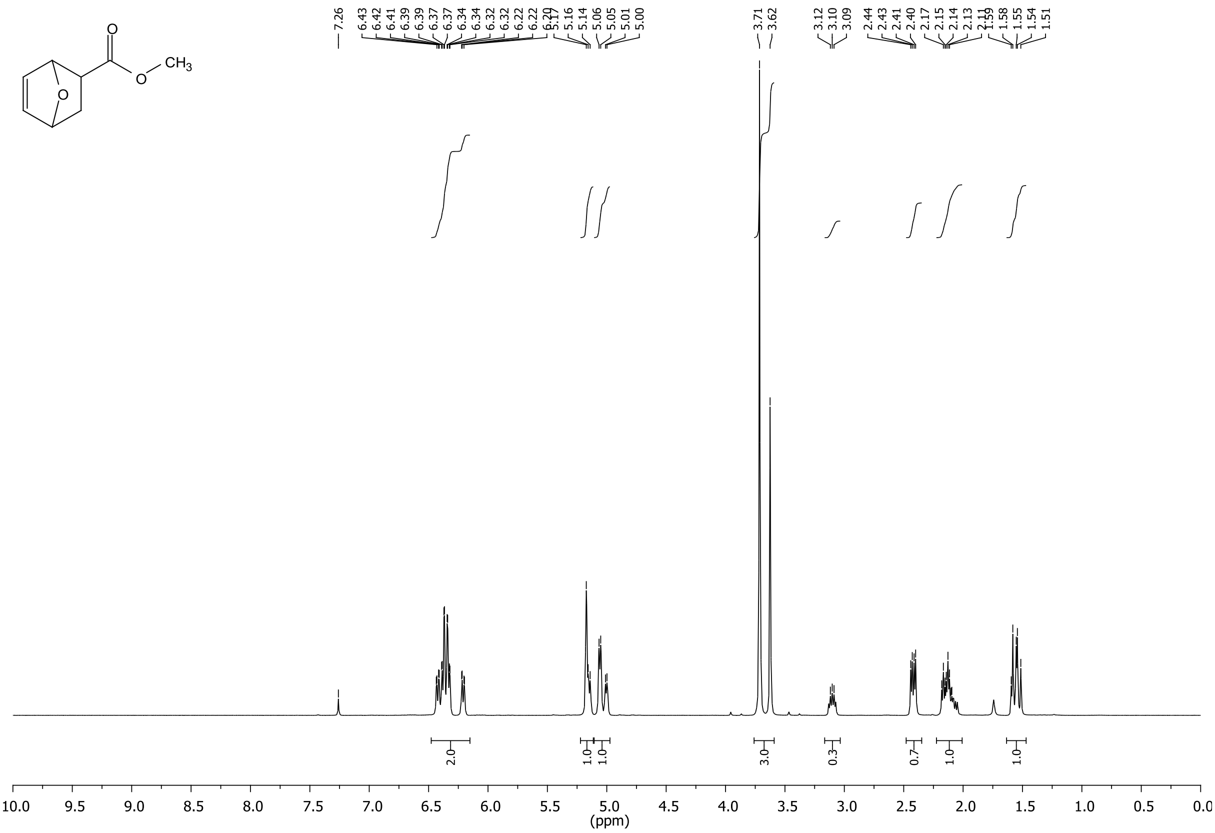

^{1}H-NMR (300.36 MHz, $CDCl_3$) of compound **3**

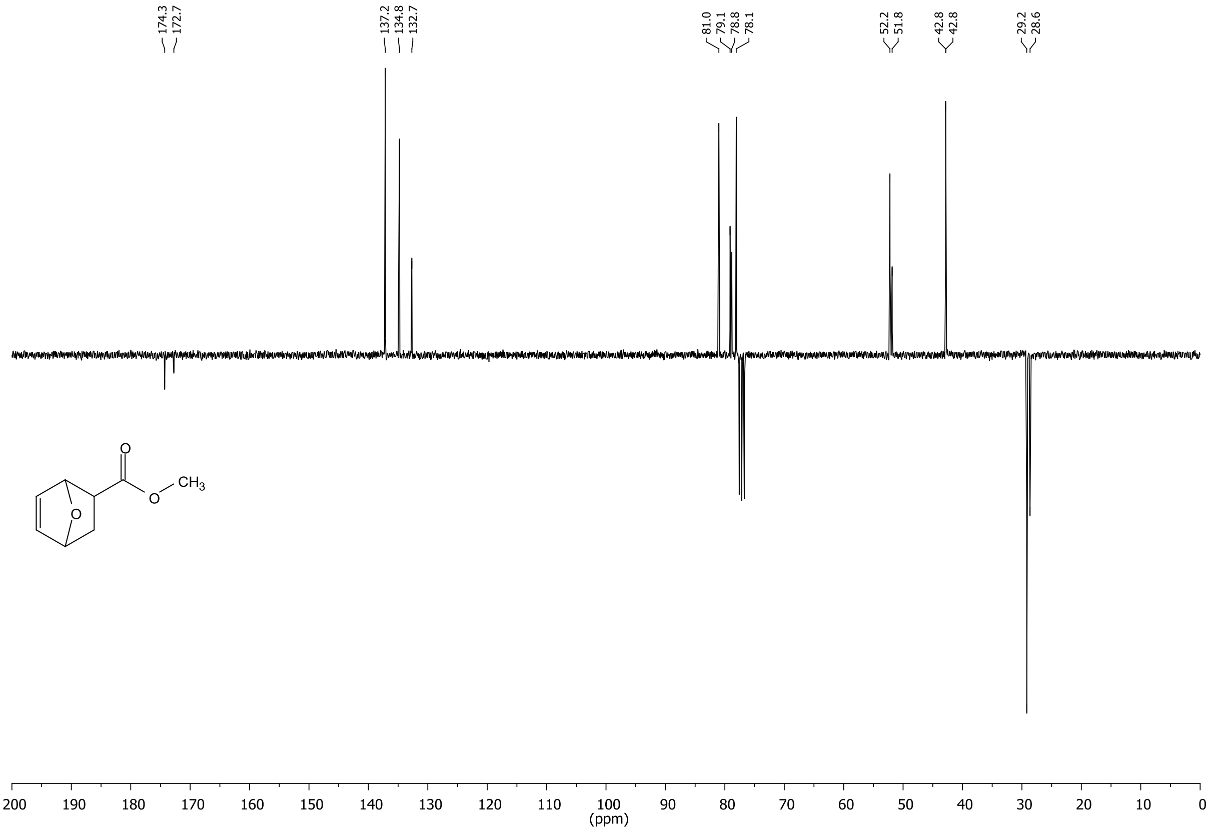

^{13}C-NMR,APT (75.53 MHz, $CDCl_3$) of compound **3**

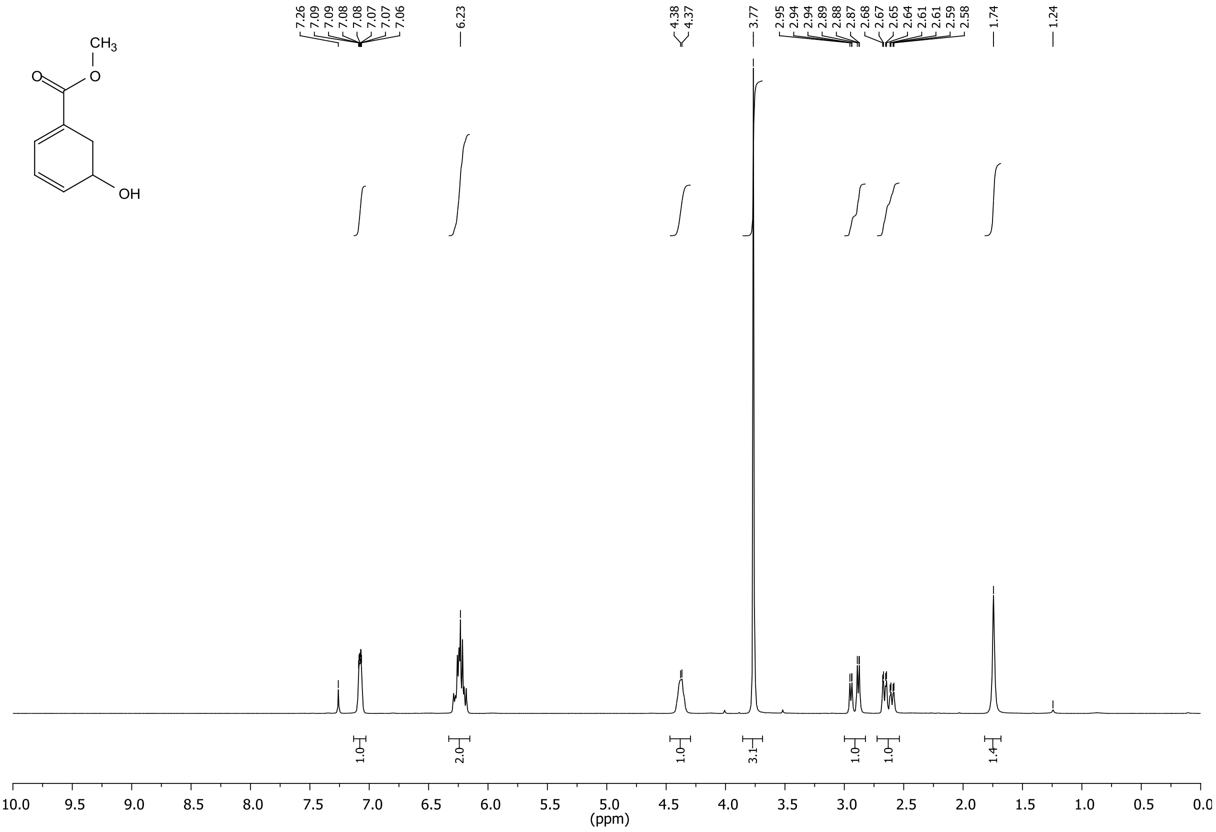

^{1}H-NMR (300.36 MHz, $CDCl_3$) of compound **4**

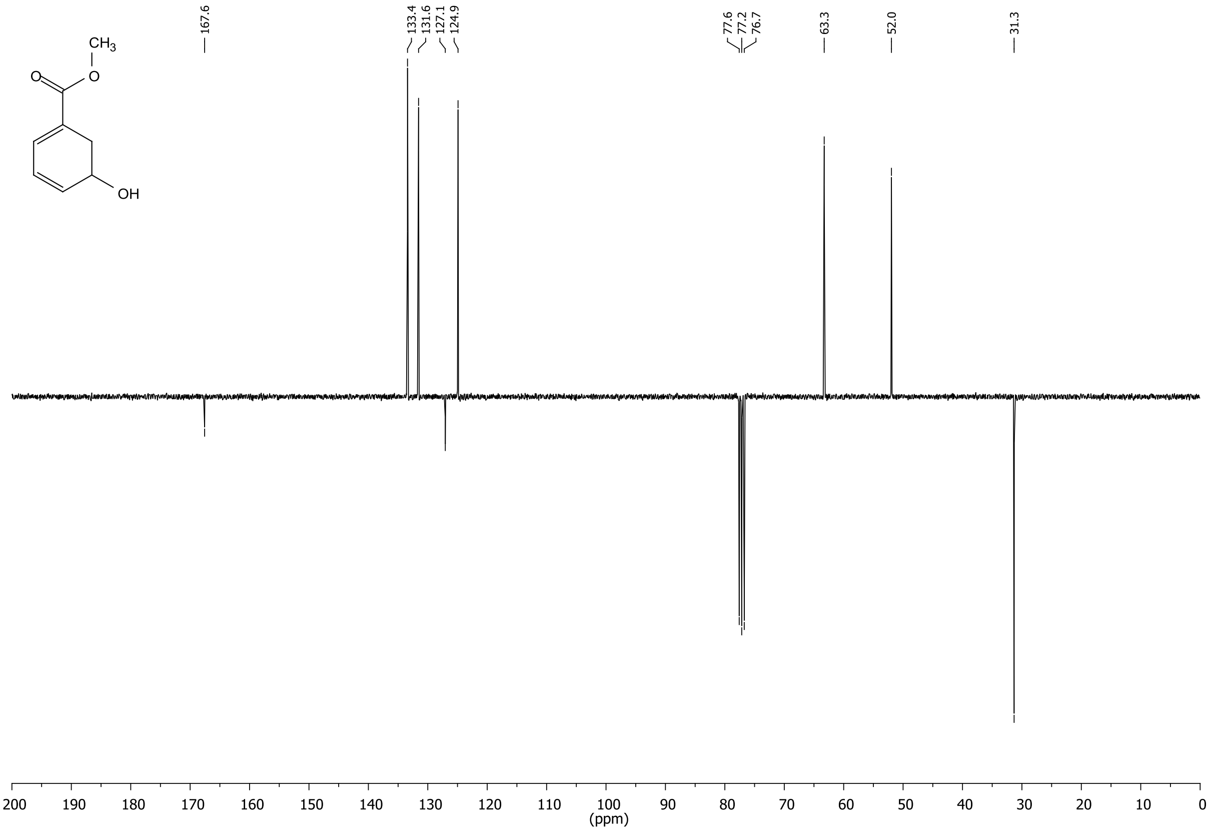

^{13}C-NMR (75.53 MHz, $CDCl_3$) of compound **4**

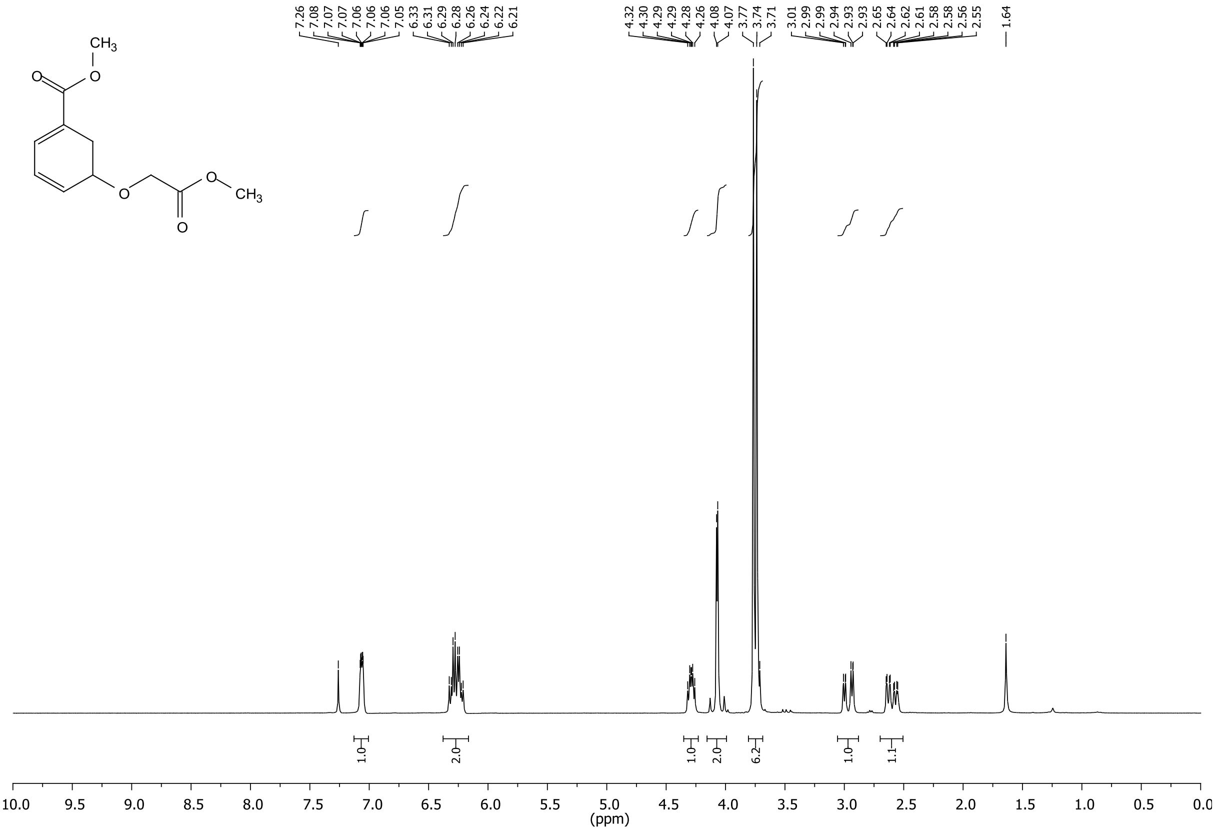

^{1}H-NMR (300.36 MHz, $CDCl_3$) of compound **5**

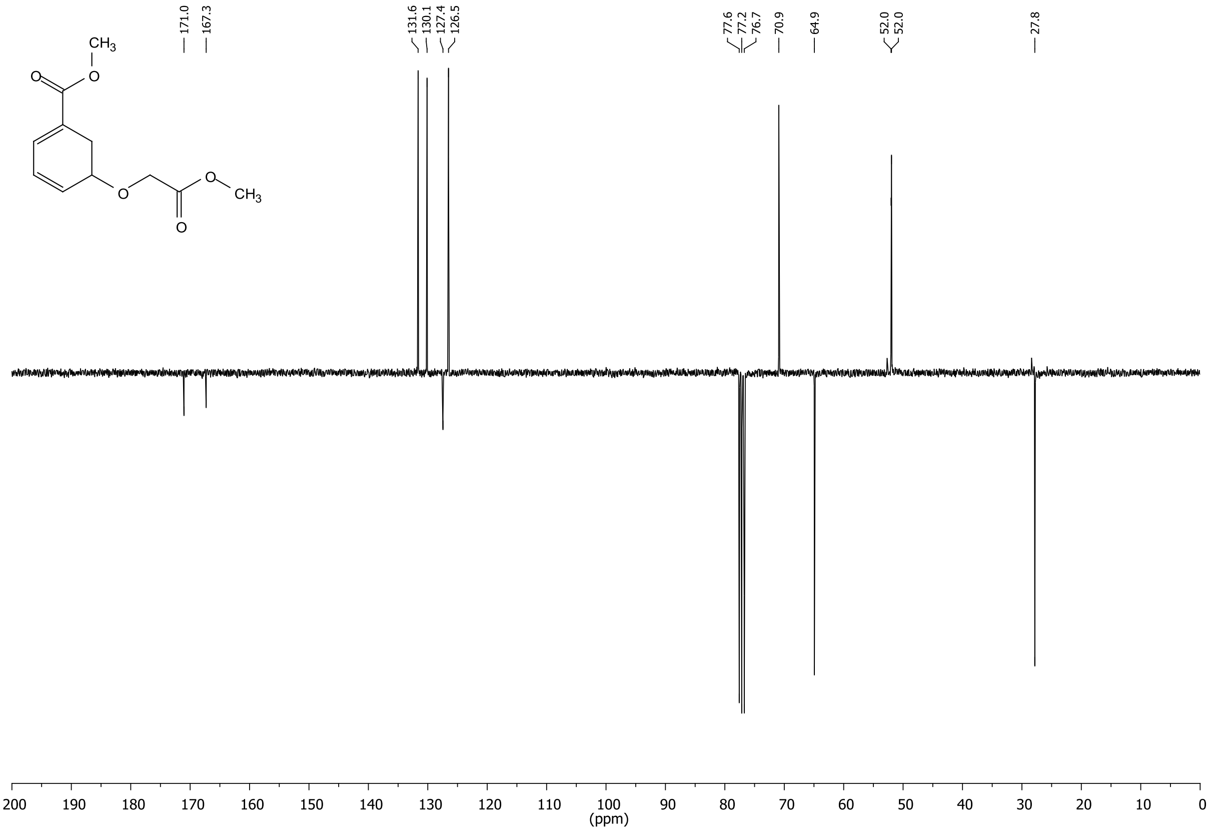

^{13}C-NMR,APT (75.53 MHz, $CDCl_3$) of compound **5**

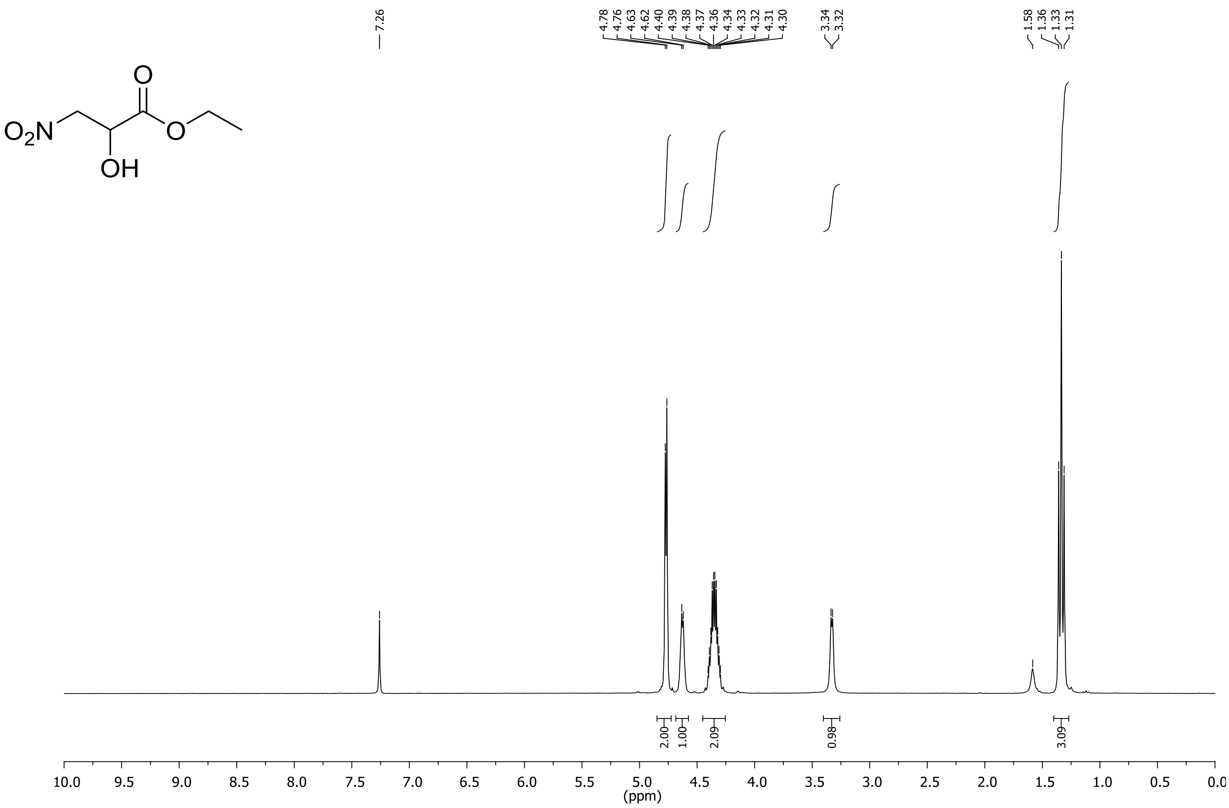

^{1}H-NMR (300.36 MHz, $CDCl_3$) of compound **6**

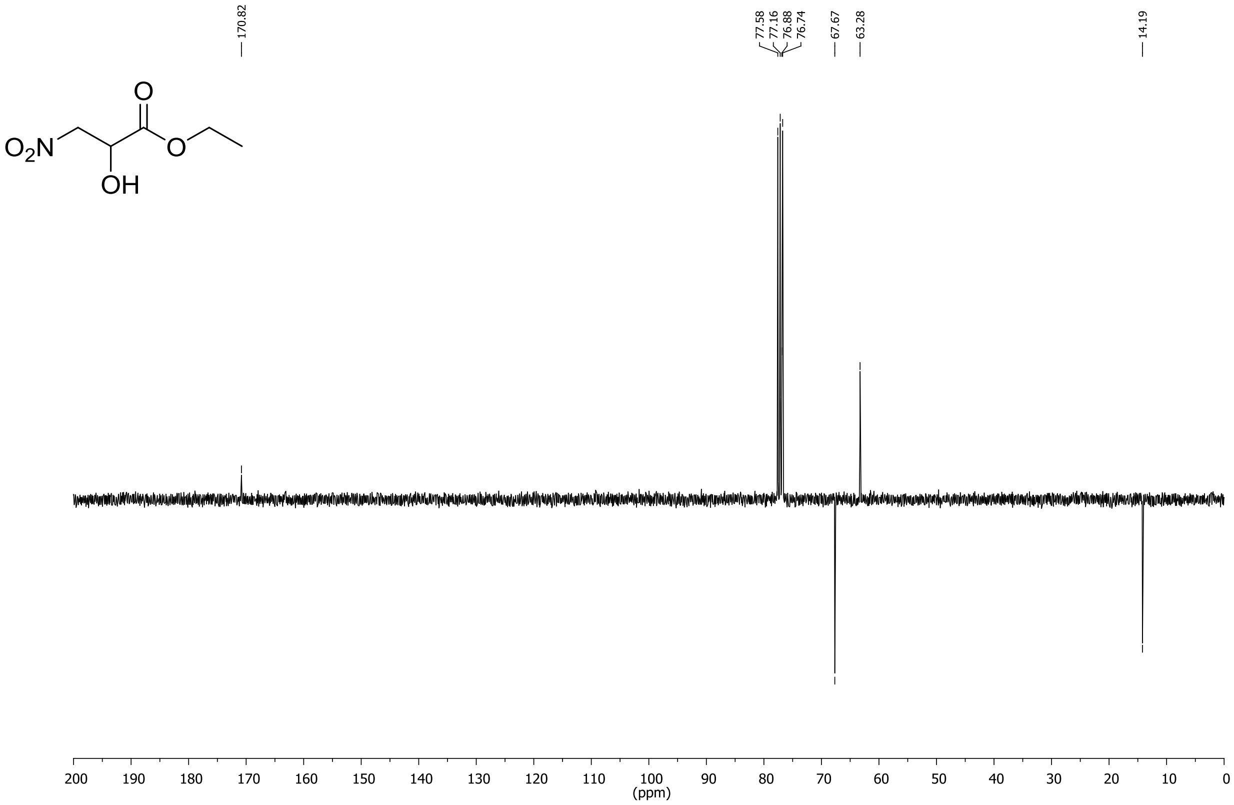

^{13}C-NMR,APT (75.53 MHz, $CDCl_3$) of compound **6**

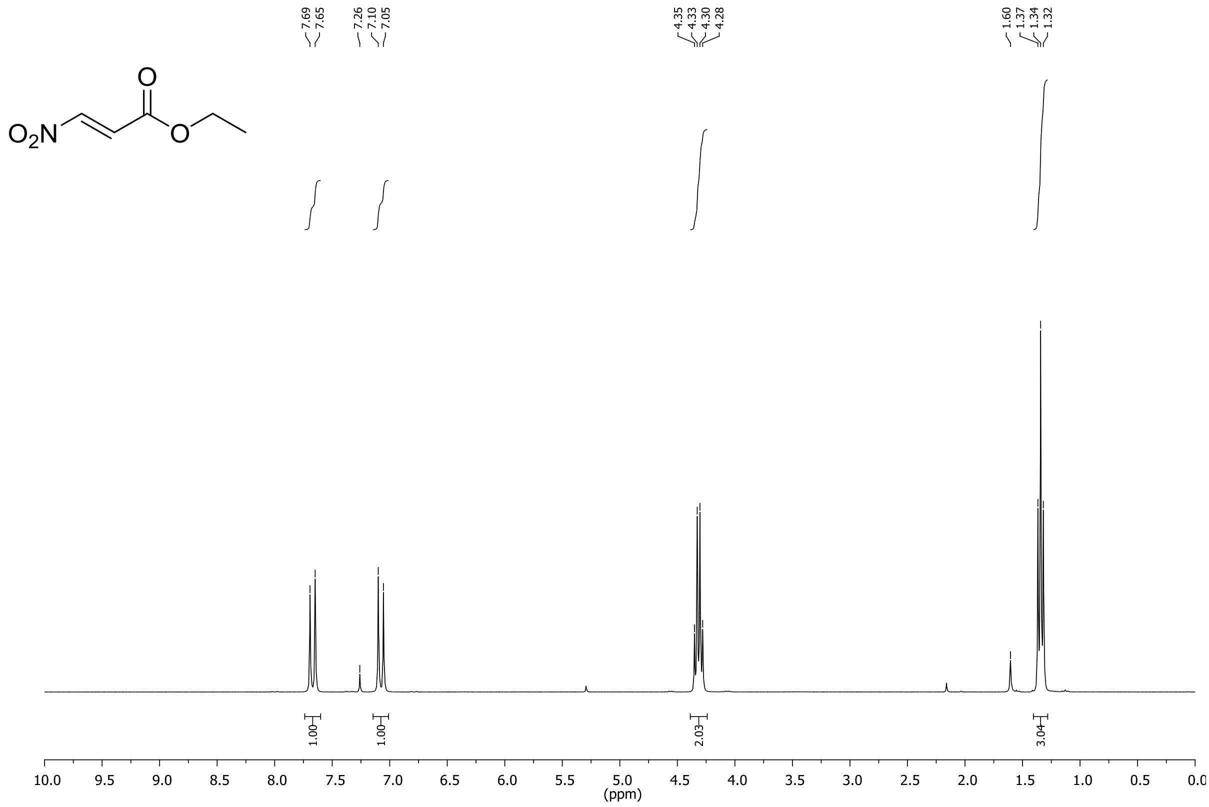

^{1}H-NMR (300.36 MHz, $CDCl_3$) of compound **7**

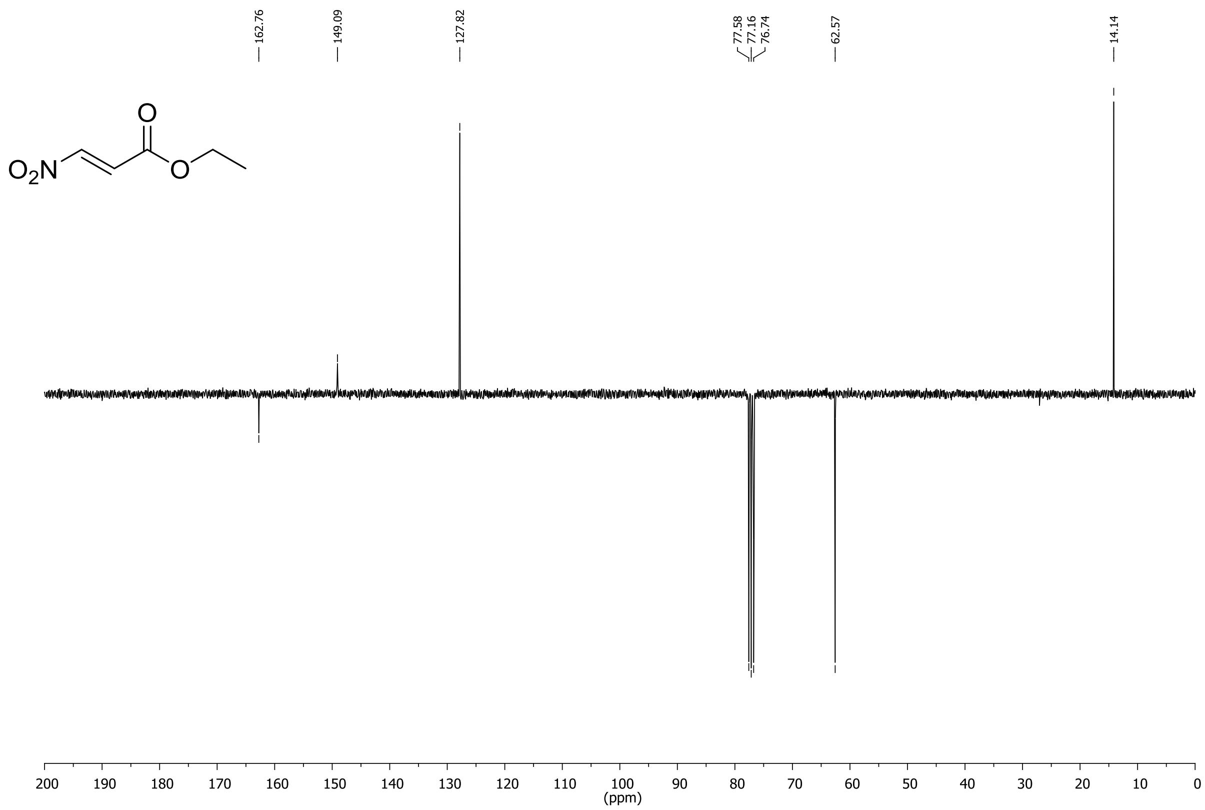

^{13}C-NMR (75.53 MHz, $CDCl_3$) of compound **7**

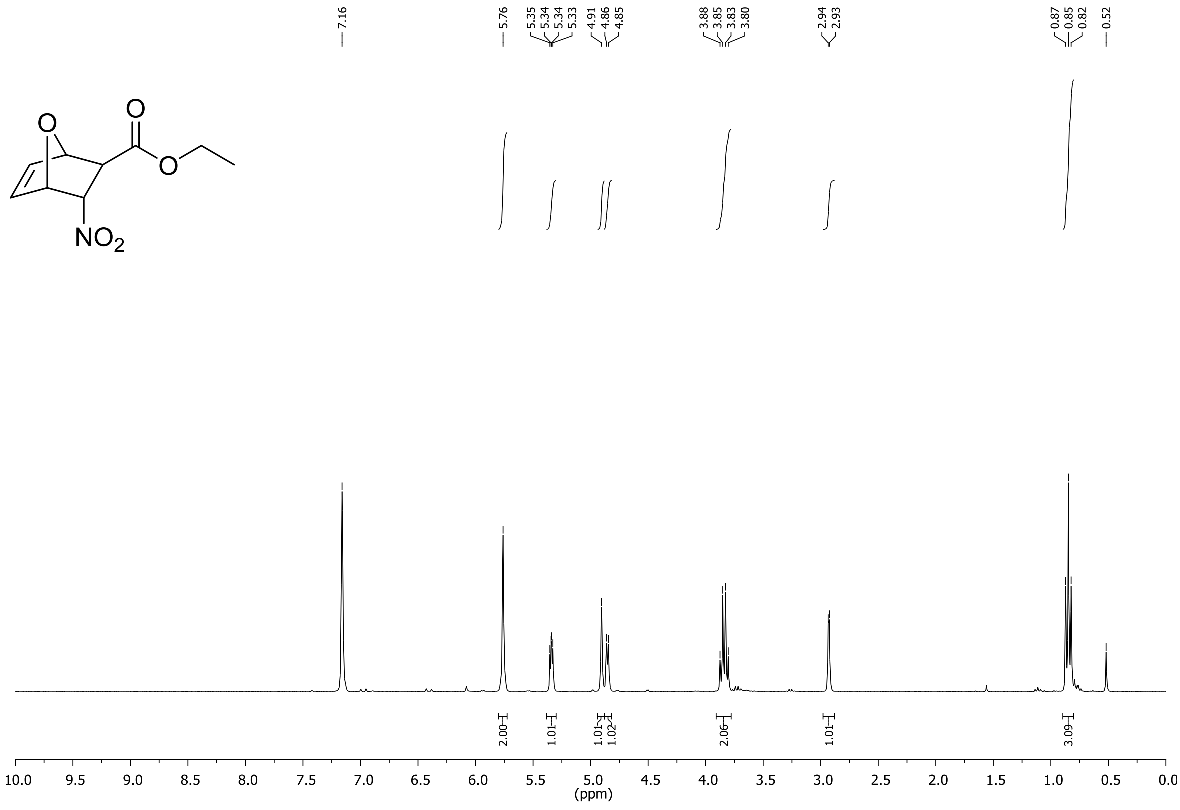

^{1}H-NMR (300.36 MHz, C_6D_6) of compound **8**

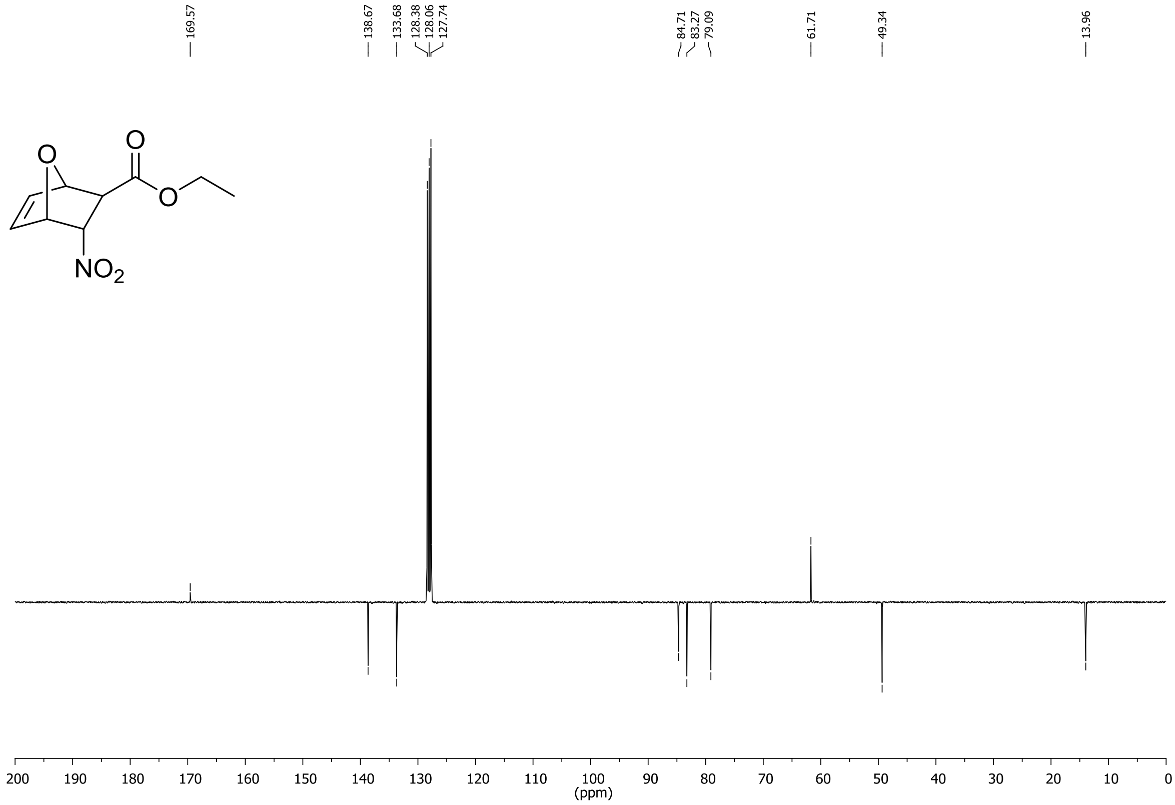

^{13}C-NMR,APT (75.53 MHz, C_6D_6) of compound **8**

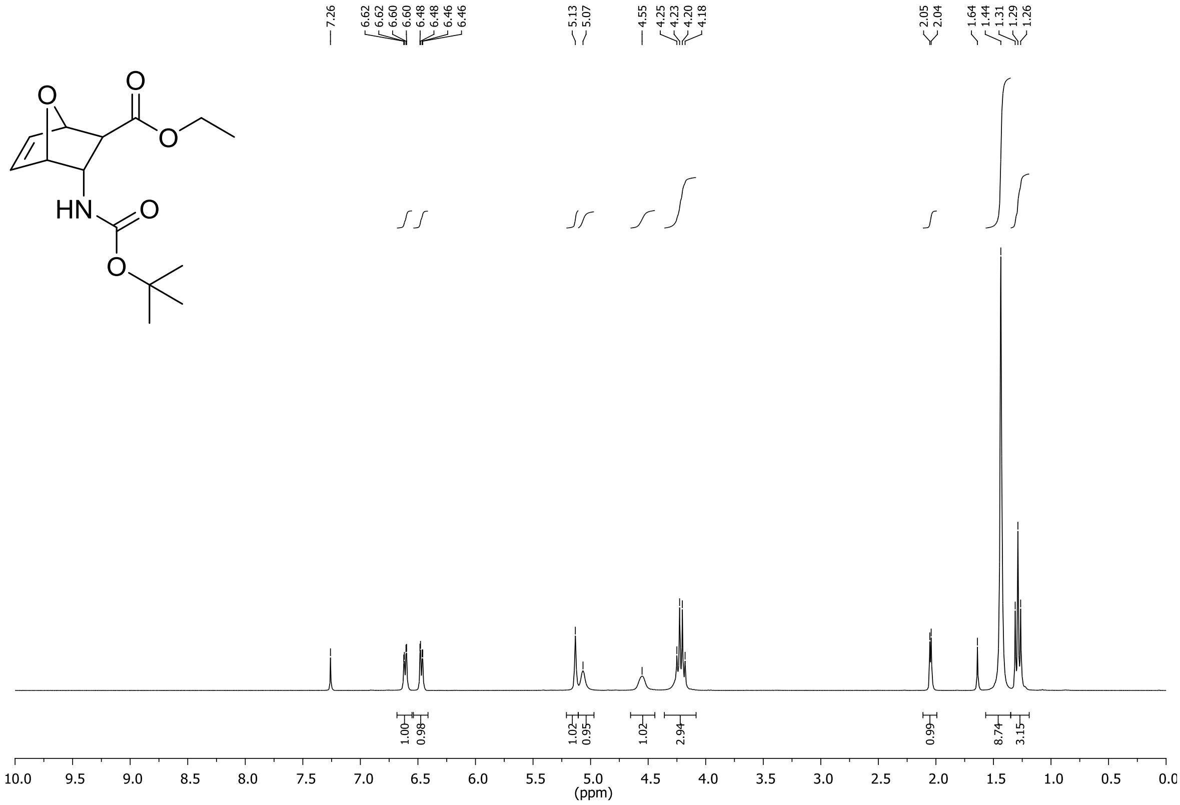

^{1}H-NMR (300.36 MHz, $CDCl_3$) of compound **9**

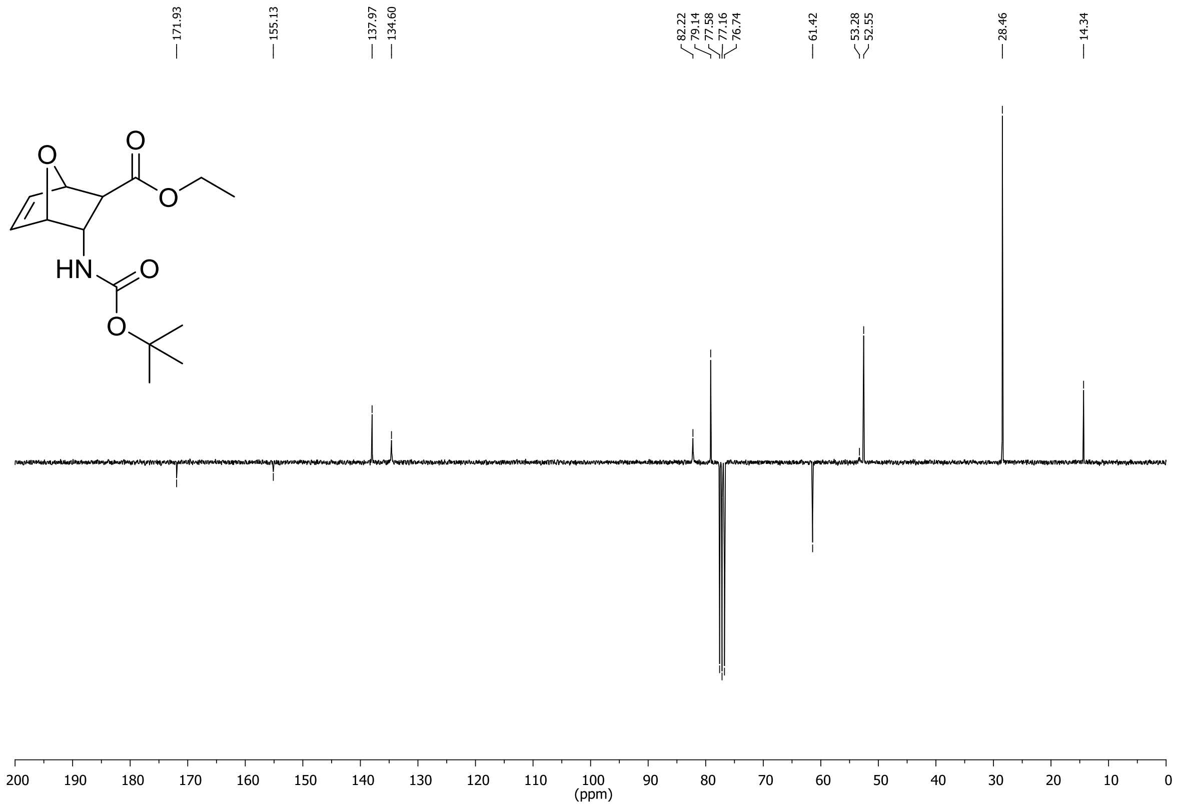

^{13}C-NMR,APT (75.53 MHz, $CDCl_3$) of compound **9**

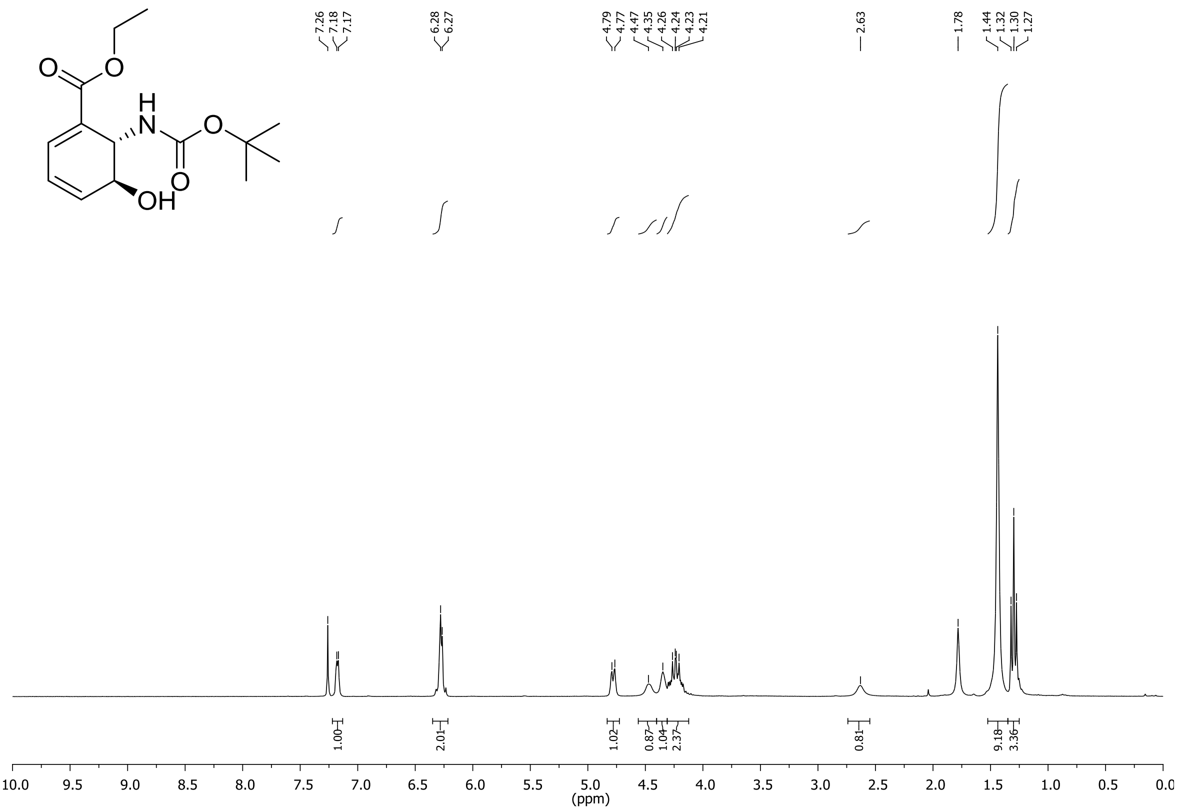

^{1}H-NMR (300.36 MHz, $CDCl_3$) of compound **10**

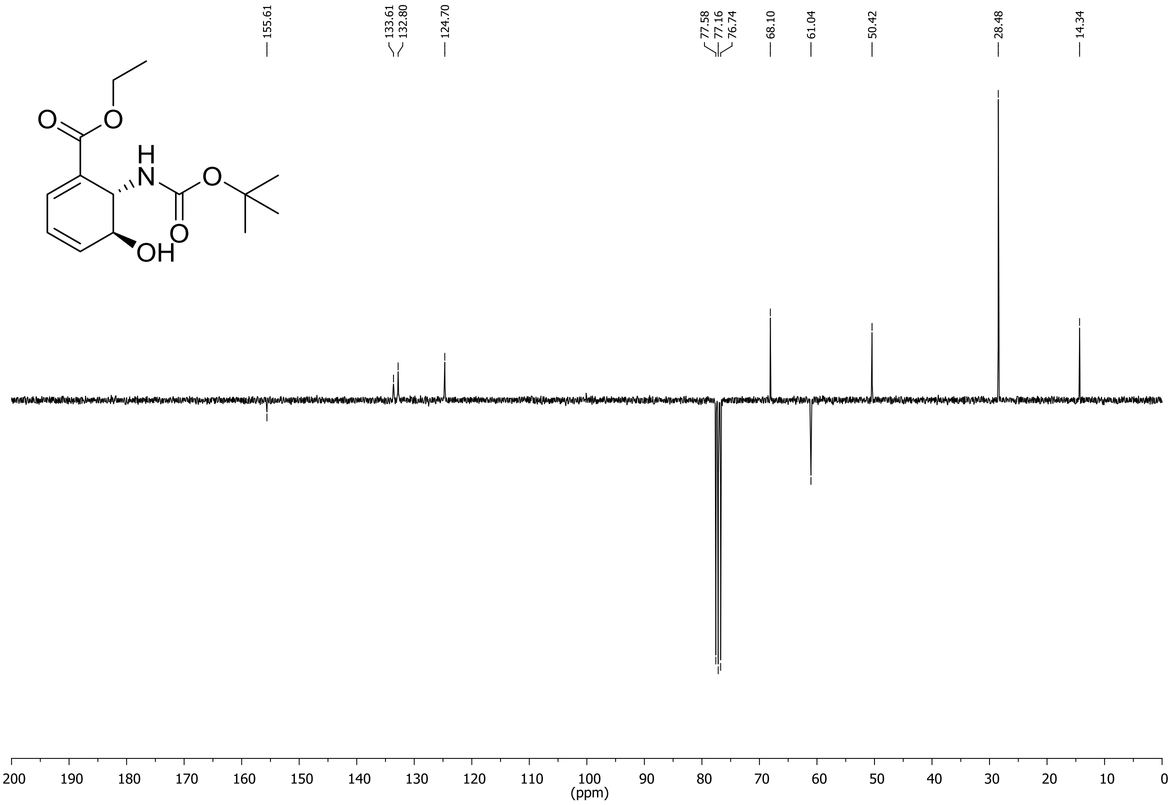

^{13}C-NMR,APT (75.53 MHz, $CDCl_3$) of compound **10**

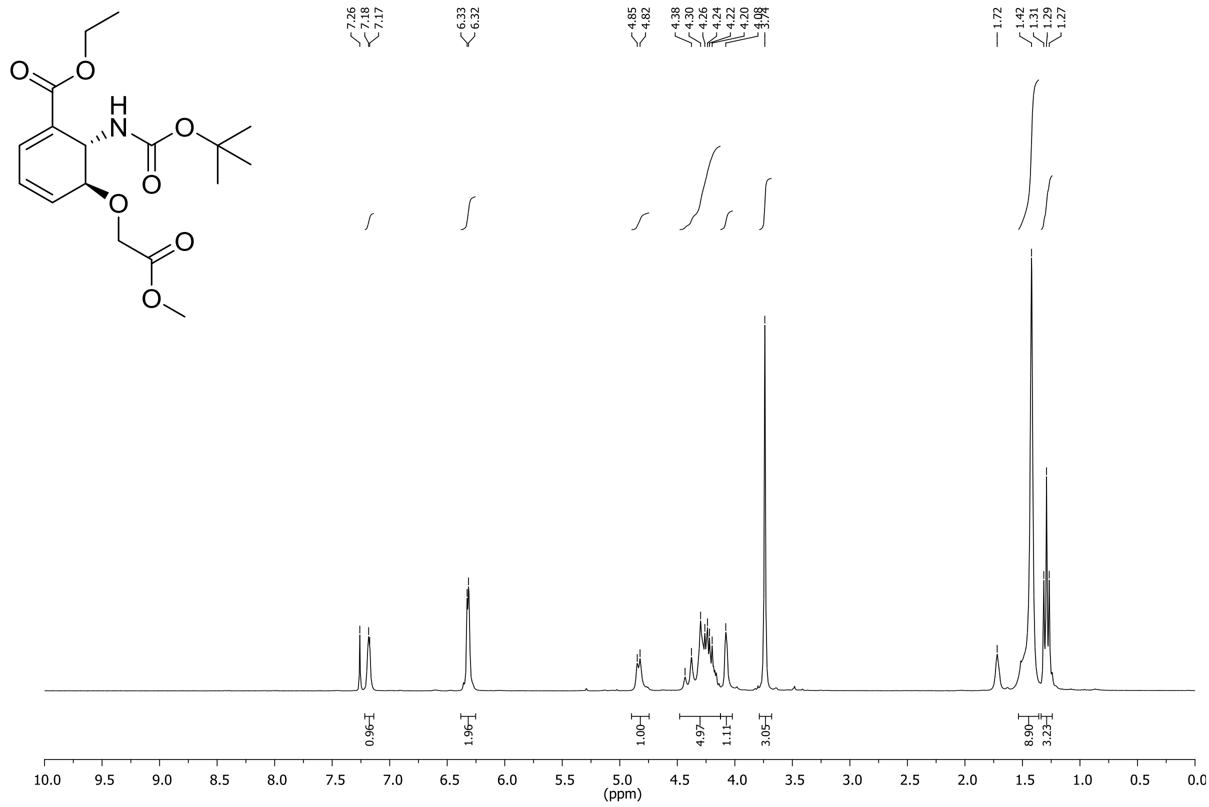

^{1}H-NMR (300.36 MHz, $CDCl_3$) of compound **11**

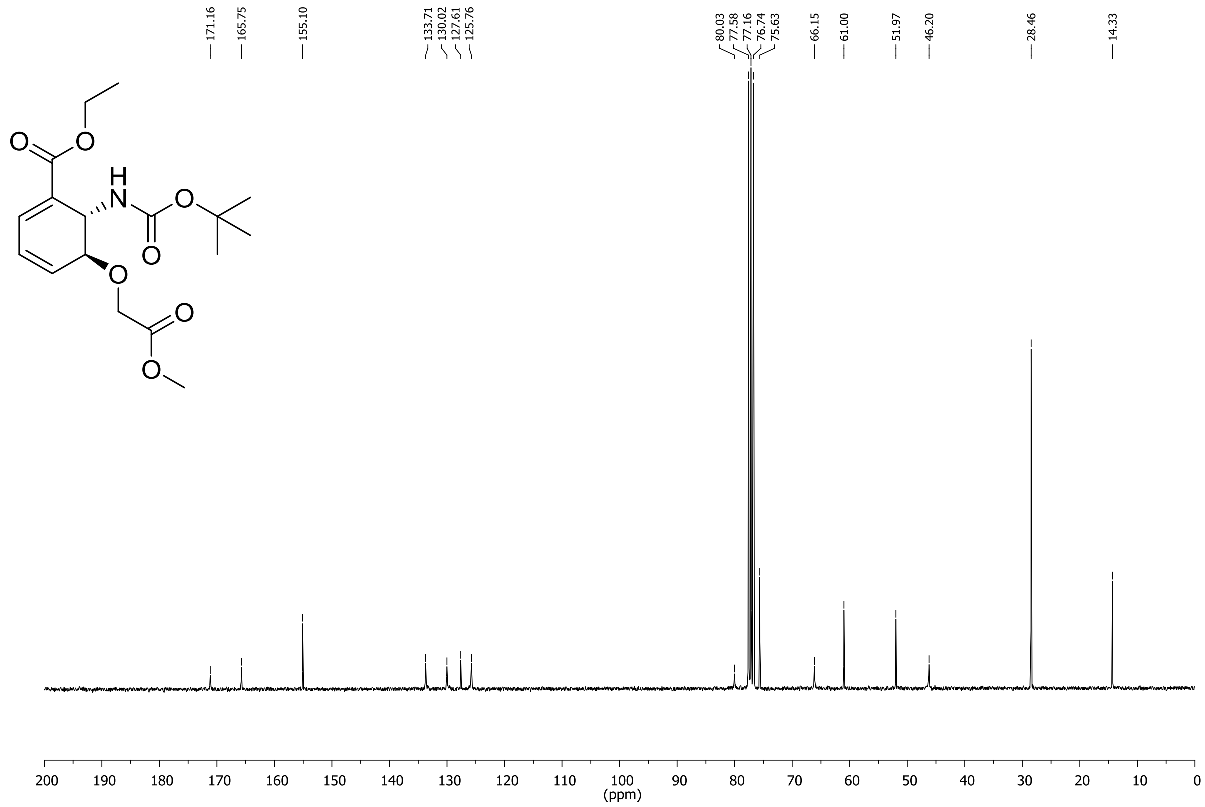

^{13}C-NMR (300.36 MHz, $CDCl_3$) of compound **11**

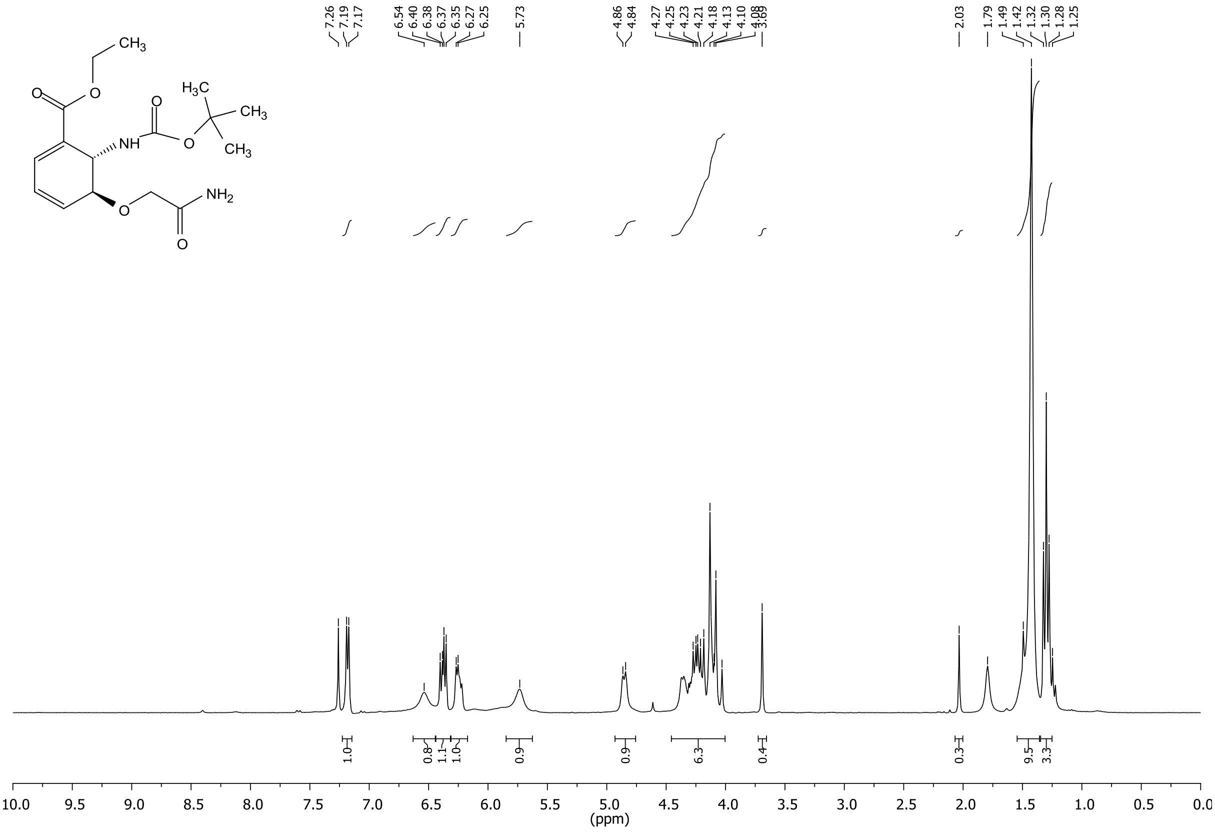

^{1}H-NMR (300.36 MHz, $CDCl_3$) of compound **12**

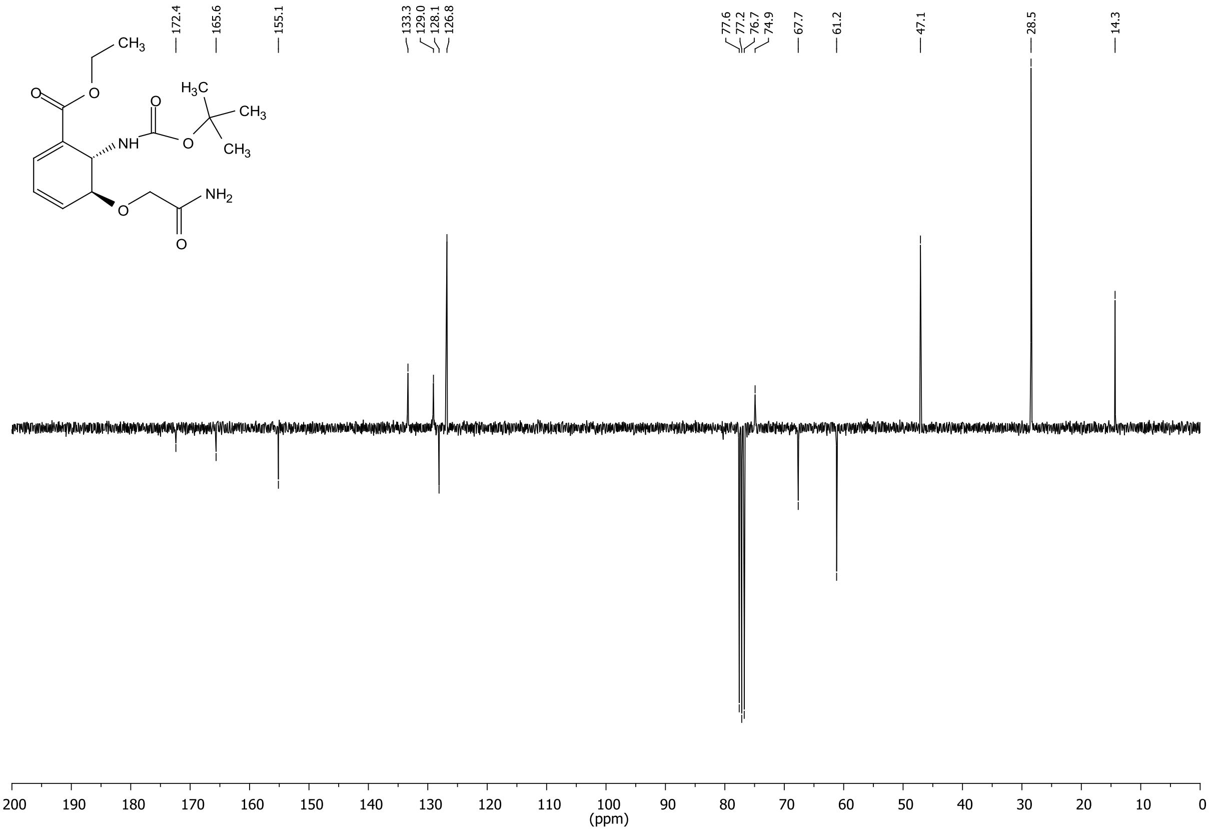

^{13}C-NMR,APT (75.53 MHz, $CDCl_3$) of compound **12**

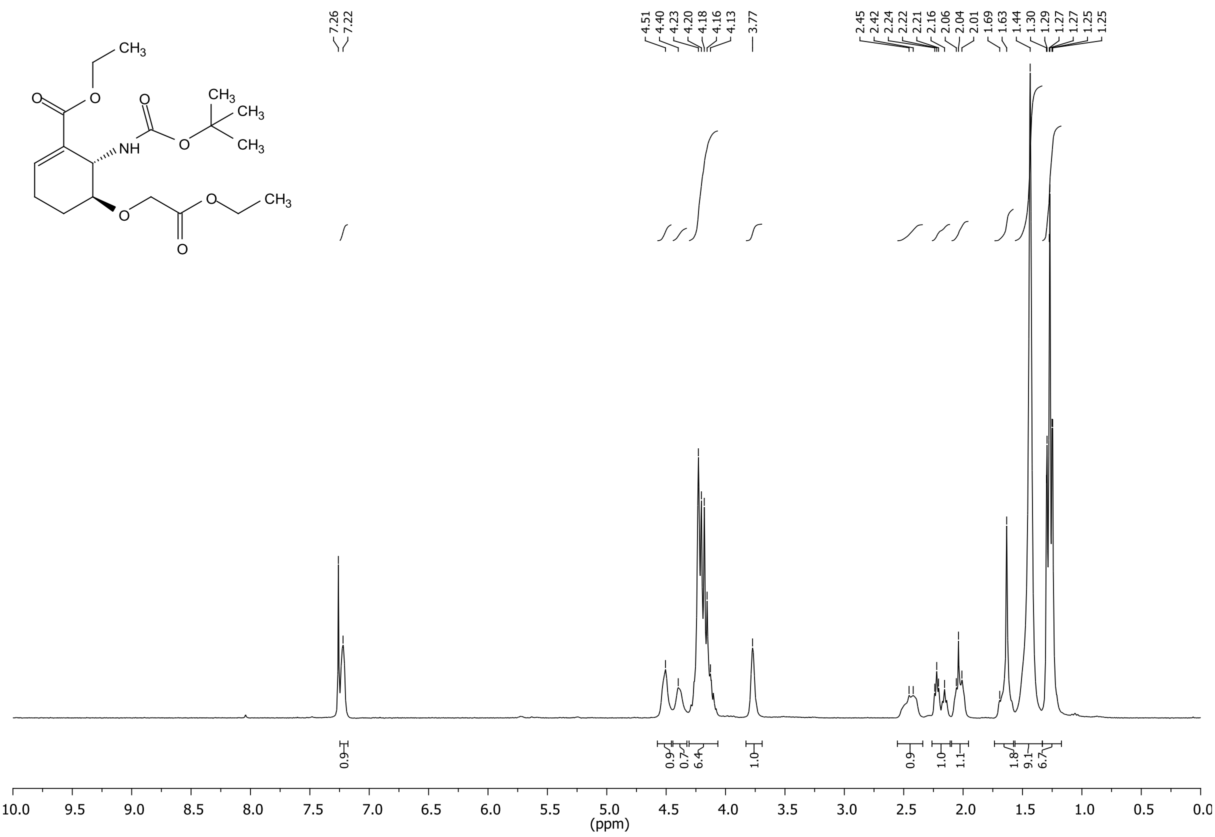

^{1}H-NMR (300.36 MHz, $CDCl_3$) of compound **13**

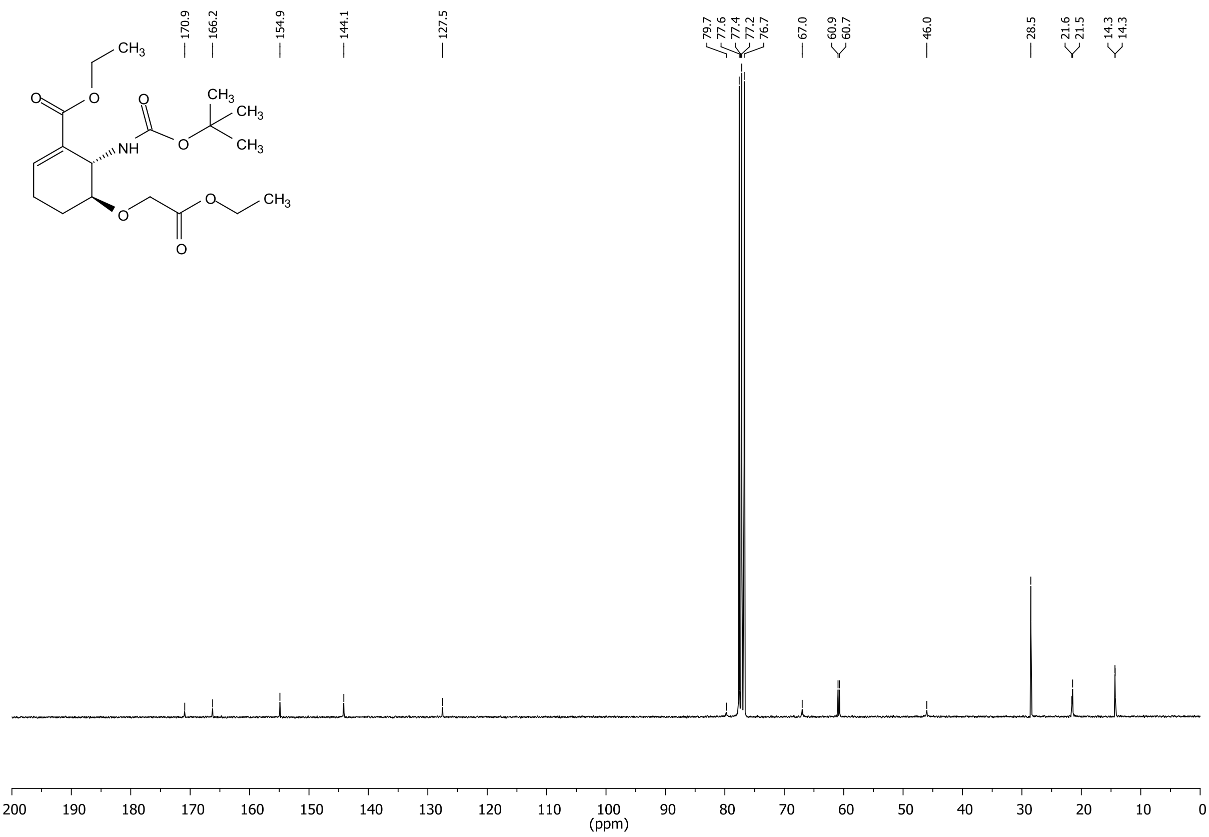

^{13}C-NMR,APT (75.53 MHz, $CDCl_3$) of compound **13**

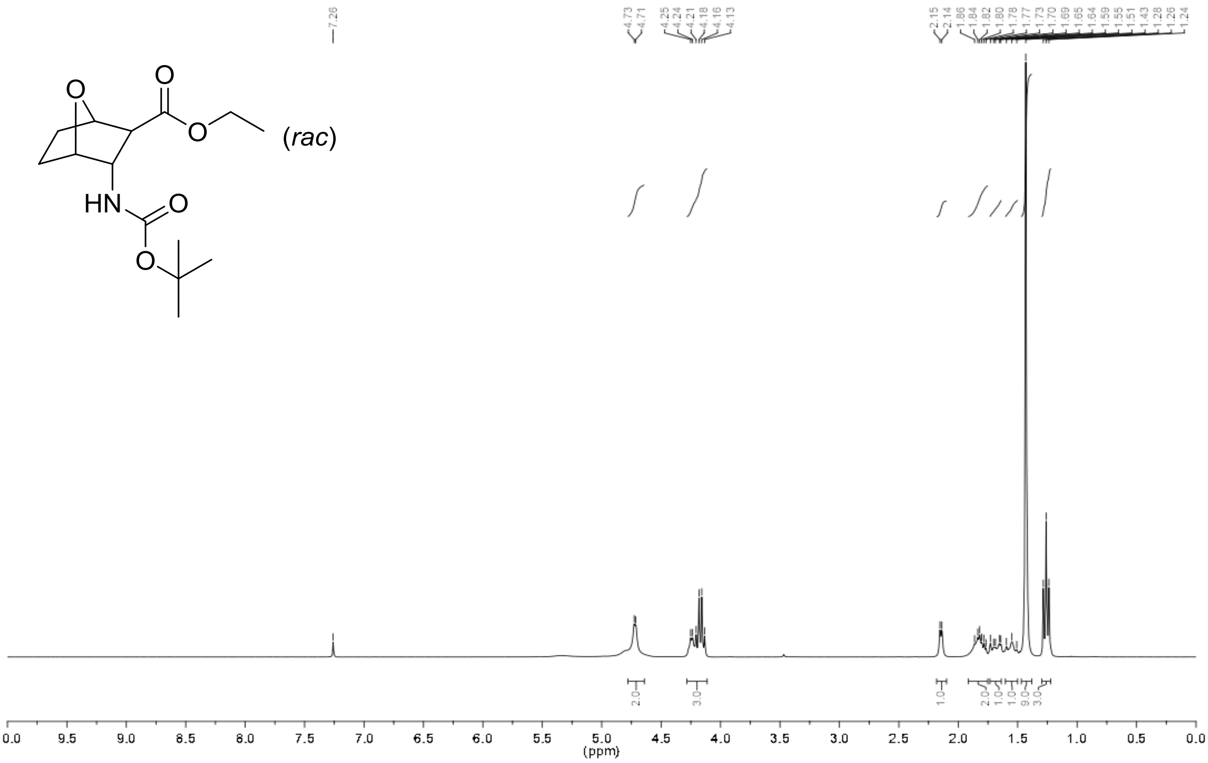

^{1}H-NMR (300.36 MHz, $CDCl_3$) of compound **14**

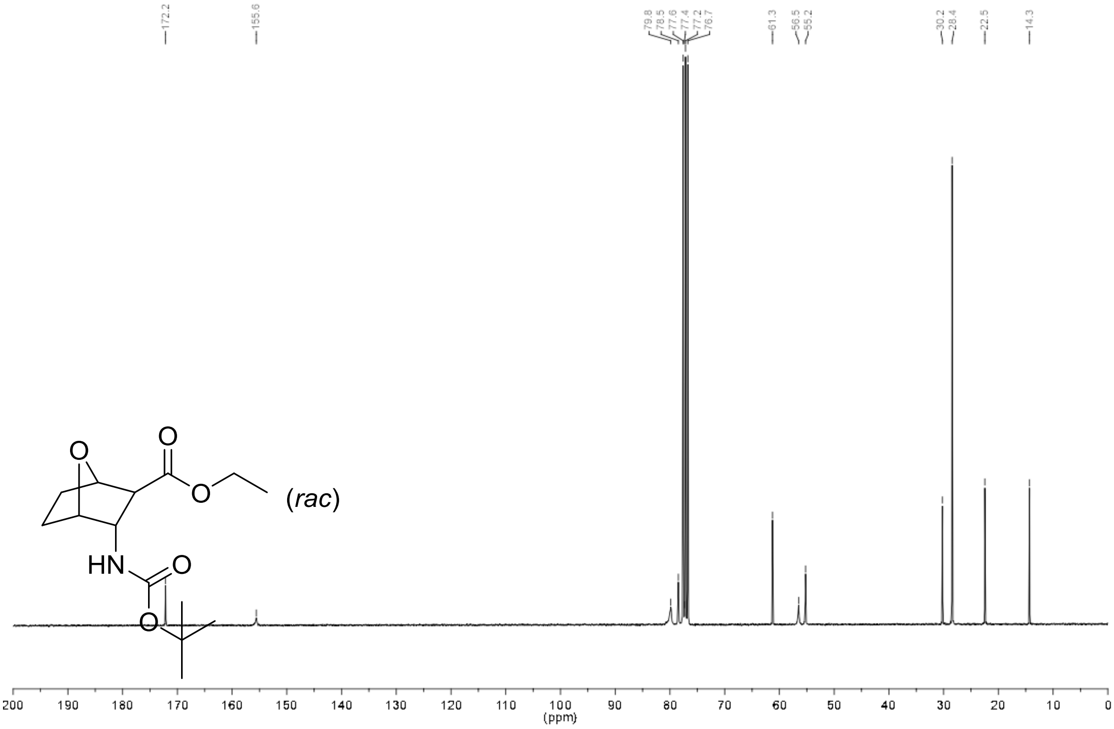

^{13}C-NMR,APT (75.53 MHz, $CDCl_3$) of compound **14**

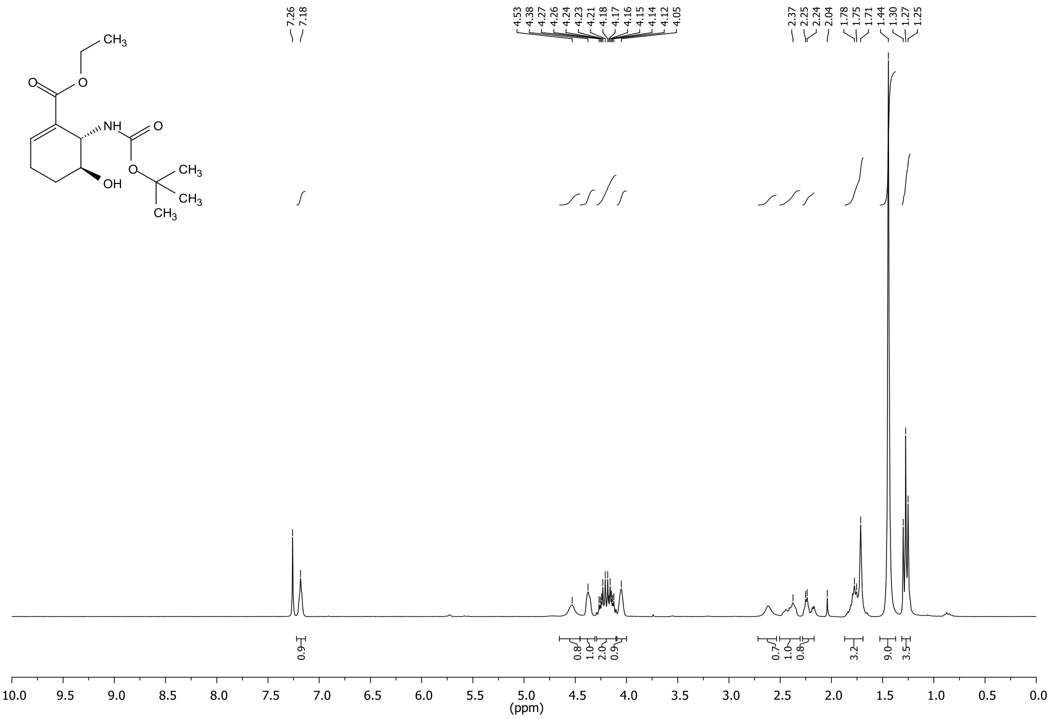

^{1}H-NMR (300.36 MHz, $CDCl_3$) of compound **15**

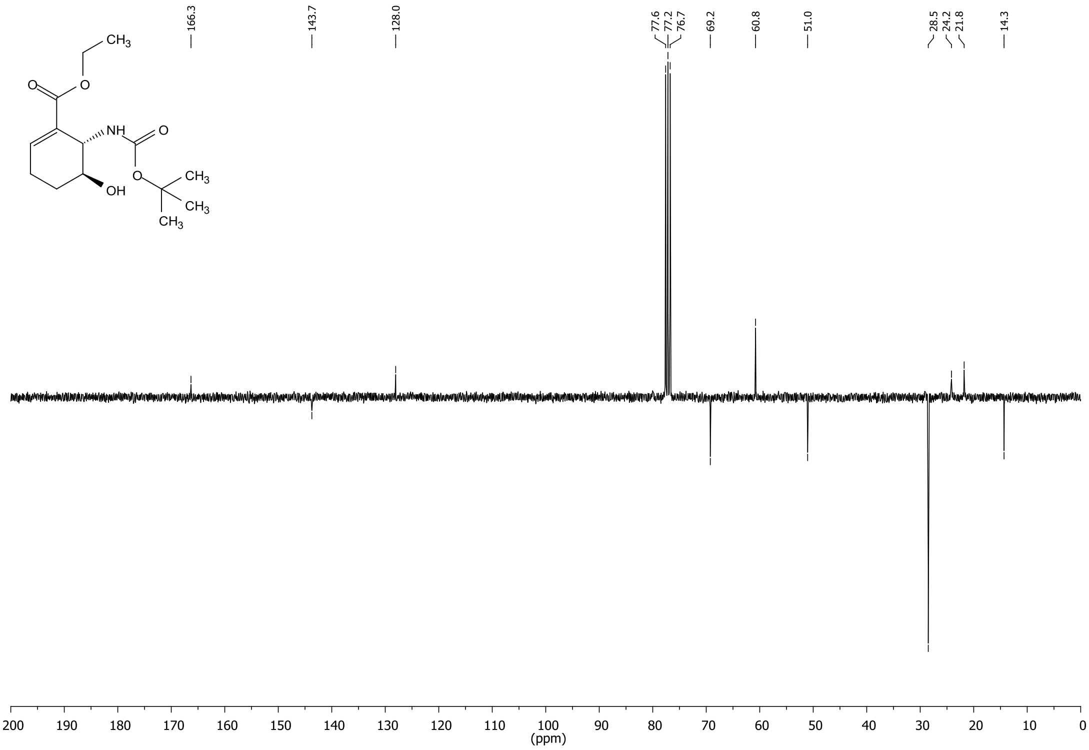

^{13}C-NMR,APT (75.53 MHz, $CDCl_3$) of compound **15**

1
